# Supplementary material for: Comparison of state-of-the-art error-correction coding for sequence-based DNA data storage
Source: Nat Commun. 2026 Mar 14;17:3963. doi: 10.1038/s41467-026-70548-3 (PMC13133123; doi:10.1038/s41467-026-70548-3)
Supplement: Supplementary file 1 — Supplementary Information [file 41467_2026_70548_MOESM1_ESM.pdf]

Supplementary Notes

Supplementary Note 1: Patches to codec implementations

In order to ensure representative error-correction performance or facilitate automated testing, the implementations of the DNA-Aeon, Goldman, HEDGES, and Yin-Yang codecs were patched. The patches are available in the GitHub repository at github.com/fml-ethz/dt4dds-benchmark, and outlined in the following.

DNA-Aeon

For DNA-Aeon, a custom codebook was used to change homopolymer constraints. In addition, the encoding script was adjusted to enable a local python runtime environment, as well as support custom paths for in- and output files. Moreover, intermediate files generated during encoding were renamed to prevent race conditions due to name clashes.

Goldman

The encoding pipeline was modified to record the total number of segments used for encoding. This information was then used in the decoding pipeline to filter out invalid segments. Moreover, the decoding pipeline was modified to support arbitrary binary files, filter out corrupted segments, as well as join overlapping segments to yield consensus sequences by majority voting prior to segment decoding.

HEDGES

The implementation at the official GitHub repository[1] has been changed to a pure C++ implementation in November 2024, from the original implementation as C++ modules callable from Python 2. Unfortunately, the old implementation has been wiped from the repository during the re-implementation. Therefore, a fork of the old implementation must now be used, such as github.com/shulp2211/hedges. This study is using the old, original implementation throughout. Individual en- and decoding scripts were set up, following the pipelines used in the provided test script. In addition, these scripts were changed to support arbitrary file sizes by padding the last packet with sentinel values. As a result, effective code rate depends on input file size, due to varying completeness of the last packet. As the decoding depends on the presence of specific adapter sequences up- and downstream of the data-carrying nucleotides, these are added and removed to all sequences during de- and encoding respectively.

Yin-Yang

A script file for implementing the en- and decoding tasks described in the README was set up to serve as the entry point for their automation. In addition, supplementary files required for decoding were saved separately after encoding and automatically re-supplied to the script during the decoding step.

Supplementary Note 2: Considerations for reporting storage density

We chose to report code rates and storage densities without the overhead of amplification adapters or other sequence components unrelated to the data encoding in this study. This means both metrics only consider the codec's sequence output incl. indexes, redundancy, etc. as the nucleotide count used for encoding. Besides simplicity, the main reasons for this were the absence of any sequence overhead in the synthetic benchmarks, as well as the low relevance to the comparison between codecs in this study (e.g., codecs had identical amplification adapters). Further complicating the matter, our *in-vitro* replication required padding the output of some codecs to achieve a homogeneous sequence length (see Supplementary Fig. 14). As experimental considerations rather than codec constraints necessitated this padding, including it in the calculations for storage density would have unduly affected codecs which produced shorter sequences.

A major downside of this choice is the comparableness outside of the codecs in this study. While this is a common problem in the DNA data storage literature due to differences in the choice of amplification adapters or the support for random access, it impedes fair comparisons. Thus, we also report our experimental storage densities and those from the literature using other definitions of the nucleotide count in Supplementary Table 11. In all cases, a molecular weight of 662 g mol^{-1} bp^{-1} (i.e., double-stranded DNA at 616 g mol^{-1} bp^{-1} with two sodium counterions at 2x23 g mol^{-1} bp^{-1}) is assumed, leading to the following equations:

$$\text{Code rate in bit nt}^{-1} = \frac{\text{File size in bit}}{\text{Nucleotide count}}$$

$$\text{Storage density in EB g}^{-1} = \frac{\text{Code rate in bit nt}^{-1} \times \frac{1}{8}\text{ byte bit}^{-1} \times 10^{-18}\text{ EB byte}^{-1}}{662\text{ g mol}^{-1}\text{ bp}^{-1} \times N_A^{-1} \times 1\text{ bp nt}^{-1} \times \text{Physical redundancy}}$$

This simplifies to:

$$\text{Storage density in EB g}^{-1} = \frac{\text{Code rate in bit nt}^{-1}}{\text{Physical redundancy}} \times 113.7\text{ EB bit}^{-1}\text{ nt g}^{-1}$$

Note that the exact molecular weight used for calculations varies slightly between studies (e.g., Organick et al.[2] use 325 g mol^{-1} nt^{-1} with single-stranded DNA and assume 1024^6 byte EB^{-1}). To harmonize the results shown in Supplementary Table 11, results from literature were re-calculated using the above definitions (i.e., using 331 g mol^{-1} nt^{-1} for single-stranded DNA and 1000^6 byte EB^{-1}). This leads to slight differences in the listed storage densities compared to those reported in the original studies.

Supplementary Note 3: Variations in sequencing depth and coverage

In the experimental replications of the low- and high-fidelity workflows, it was assumed that each codec's sequences were homogeneously represented in the oligo pools, if they were synthesized simultaneously. To ensure this, the order of the sequences supplied to the commercial synthesis companies was randomized, thereby precluding any chip-related bias.[3] Nonetheless, the sequencing data strongly suggests the presence of a systematic bias between codecs, highlighted by the inhomogeneity of sequencing depths (see Supplementary Fig. 15).

As outlined in the main manuscript, it is inconclusive which process (i.e., synthesis, amplification, or sequencing) specifically caused this bias. However, given the randomization during synthesis (see above) and the presence of this bias in both synthesis technologies, it is unlikely to be related to synthesis. PCR is known to have biased amplification,[3–6] which would explain the difference in the bias's severity between the low- and high-fidelity scenario (due to different numbers of PCR cycles in the two workflows).

Plausible causes for the PCR-induced bias are sequence features introduced by codecs (e.g., repetitive elements in the Goldman codec, indexing regions) or the barcodes added to each codecs' sequences (see Supplementary Fig. 14 and Supplementary Table 10). More experiments would be needed to conclusively elucidate the origin of the inhomogeneity. However, the observed bias correlates strongly with the rate of sequence loss in the sequencing data (see Supplementary Fig. 15), thereby likely causing the systematic deviations between simulated predictions and the experimental results. Specifically, amplification after synthesis likely caused specific enrichment of sequences from some codecs in the oligonucleotide pools (see Sequencing depth of 1000x samples in Supplementary Fig. 15). Then, during dilution, enriched sequences were more likely to be sampled. This results in a lower probability for sequence dropout during sequencing, reducing the need for logical redundancy for the decoder.

Supplementary Tables

Supplementary Table 1: Full results of clustering performance. For each clustering algorithm, multiple parameter sets were tested, if supported. These parameter sets deviated from the default settings in the parameters described in the Parameters column. Each parameter set was tested once with experimental data from electrochemical synthesis (Elec.), and once with experimental data from synthesis by material deposition (M.D.), see Methods for additional details and definitions of sensitivity, accuracy, and specificity. The best-performing parameter set of each clustering algorithm is highlighted with green shading. Note that MMseqs2 was not suited for further consideration and pairing with codecs due to common violation of the memory constraint.

Clustering	Parameters	Scenario	Sensitivity	Accuracy	Specificity	Time / s
Naïve	None	Elec.	1.000	0.997	0.057	3
		M.D.	1.000	1.000	0.084	2
CD-Hit	Default	Elec.	1.000	0.999	0.334	161
		M.D.	1.000	1.000	0.895	145
	Identity threshold 80%<br>Word size 5	Elec.	1.000	0.999	0.959	4152
		M.D.	1.000	1.000	0.996	3859
	Identity threshold 85%<br>Word size 6	Elec.	1.000	0.999	0.893	278
		M.D.	1.000	1.000	0.990	243
Clover	Depth 10	Elec.	0.381	0.953	0.611	207
		M.D.	0.295	0.965	0.618	181
	Depth 15<br>Horizontal drift 5	Elec.	0.987	0.998	0.656	195
		M.D.	0.981	1.000	0.992	153
	Depth 15<br>Vertical drift 4	Elec.	0.988	0.999	0.657	179
		M.D.	0.987	1.000	0.994	151
	Depth 20	Elec.	0.990	0.999	0.594	192
		M.D.	0.998	1.000	0.993	145
	Depth 20<br>Horizontal drift 5	Elec.	0.990	0.999	0.594	217
		M.D.	0.998	1.000	0.993	149
	Depth 20<br>Vertical drift 4	Elec.	0.990	0.999	0.593	203
		M.D.	0.997	1.000	0.993	152
	Default	Elec.	0.988	0.999	0.654	189
		M.D.	0.989	1.000	0.994	144
LSH	Default	Elec.	0.987	0.999	0.190	770
		M.D.	0.997	1.000	0.443	455
MMseqs2	Cov. mode 1	Elec.	1.000	0.999	0.637	178
		M.D.	1.000	1.000	0.999	174
	Default	Elec.	1.000	0.999	0.584	196
		M.D.	1.000	1.000	0.982	162
	50% minimum identity	Elec.	1.000	0.999	0.584	184
		M.D.	1.000	1.000	0.982	174
Starcode	Default	Elec.	1.000	0.997	0.076	489
		M.D.	1.000	1.000	0.450	133
	Sphere clustering	Elec.	1.000	0.999	0.227	661
		M.D.	1.000	1.000	0.895	149
	Sphere clustering<br>Distance 6	Elec.	1.000	0.999	0.276	1710
		M.D.	1.000	1.000	0.903	194

Supplementary Table 2: Overview of codecs for DNA data storage in the literature.[7,8] Note that the cut-off date for consideration was October 2023.

Selected?	Name	Year	Inner EC	Outer EC	Constraints	In-vitro?	Code?	Ref.	Comments
	Church et al.	2012	None	None	HP	Yes	Yes	9	No error-correction component
Yes	Goldman et al.	2013	Parity	Repetition	HP	Yes	Yes	10	
	Grass et al.	2015	RS	RS	GC, HP	Yes		11	Superseded by DNA-RS
	Yazdi et al.	2015	None	None	GC, ΔG	Yes		12	Uses ultra-long sequences
	Bornholt et al.	2016	Parity	XOR/Repetition	None	Yes		13	No implementation available
	Blawat et al.	2016	BCH	RS	GC, HP	Yes		14	No implementation available
	Yazdi et al.	2017	BCH	None	GC	Yes	Yes	15	Uses ultra-long sequences
Yes	DNA Fountain	2017	RS	Fountain	GC, HP	Yes	Yes	16	
	Organick et al.	2018	None	RS	HP	Yes		17	No implementation available
	Oligoarchive	2019	Parity	Repetition	GC, HP	Yes		18	Specific to database structures
	RA code	2019	CRC	RA	GC, HP	Yes		19	No implementation available
	Large LDPC	2019	LDPC		None	Yes	Yes	20	Employs single block code
	Deng et al.	2019	LDPC	None	GC, HP			21	No implementation available
	Wang et al.	2019	None	None	GC, HP			22	No implementation available
	Anavy et al.	2019	RS	Fountain	GC, HP	Yes	Yes	23	Uses degenerate sequences
	Choi et al.	2019	None	RS	HP	Yes		24	Uses degenerate sequences
Yes	HEDGES	2020	HEDGES	RS	GC, HP	Yes	Yes	25	
Yes	DNA-RS	2020	RS	RS	None	Yes	Yes	26,27	
	Lenz et al.	2020	multiple	LDPC	None			28	No implementation available
	JPEG	2021	Parity	None	GC, HP	Yes		29	Specific to image storage
	Chen et al.	2021	LDPC	None	GC	Yes		30	Designed for artificial chromosome
Yes	Yin-Yang	2022	None	None	GC, HP, ΔG	Yes	Yes	31	
	DBGPS	2022	CRC	Fountain	HP, kmer	Yes	Yes	32	Intended for strand reconstruction
	2DDNA	2022	None	LDPC	GC, HP	Yes	Yes	33	Uses backbone for encoding
Yes	DNA-Aeon	2023	AC-based	Fountain	GC, HP, motifs	Yes	Yes	34	
	MAFFT	2023	MSA	None	None		Yes	35	Uses only multiple-sequence alignment
	Zan et al.	2023	MSA	None	GC, HP		Yes	36	No in-vitro experiment
	Zhao et al.	2024	HEDGES	RS	GC, HP	Yes	Yes	37	Uses degenerate sequences, for nanopores

Supplementary Table 3: Full results of all codec-clustering combinations. The best-performing parameter set of each clustering algorithm was paired with each codec in the basic error scenario, yielding the error rate at which decoding succeeded after clustering with 95% probability. For each codec and code rate, the best-performing clustering algorithm was selected for all further studies, and is indicated by green shading.

Codec	Code rate	Naive	CD-Hit	Clover	LSH	Starcode
	bit nt^{-1}	Default	85% identity	D15V4	Default	Sphere, distance 6
DNA-Aeon	0.50	0.002	0.066	0.029	0.024	0.014
	1.00	0.003	0.074	0.042	0.035	0.015
	1.50	0.002	0.077	0.042	0.037	0.017
DNA Fountain	0.50	0.017	0.032	-	-	0.045
	1.00	0.016	0.027	-	-	0.042
	1.50	0.010	0.051	-	-	-
DNA-RS	0.50	0.033	0.119	0.107	0.107	0.046
	1.00	0.022	0.119	0.091	0.107	0.048
	1.50	0.017	0.103	0.086	0.091	0.043
Goldman	0.34	0.016	-	-	0.067	0.023
HEDGES	0.63	0.077	0.109	-	0.133	0.086
	1.07	0.022	0.120	-	0.103	0.049
Yin-Yang	1.85	-	0.042	-	-	-

Supplementary Table 4: Sequence recovery after clustering 30 perfect reads per reference sequence for each codec-clustering pairing.

Codec	Code rate	Naïve	CD-HIT	Clover	LSH	Starcode
	bit nt^{-1}	%	%	%	%	%
DNA-Aeon	0.50	100	100	98.2	100	100
	1.00	100	100	98.6	100	100
	1.50	100	100	99.2	100	100
DNA Fountain	0.50	100	99.7	97.8	100	100
	1.00	100	99.9	98.6	100	100
	1.50	100	100	99.1	100	100
DNA-RS	0.50	100	100	98.4	100	100
	1.00	100	100	98.2	100	100
	1.50	100	100	98.2	100	100
Goldman	0.34	100	98.6	79.1	96.3	100
HEDGES	0.63	100	96.5	8.1	99.4	98.0
	1.07	100	99.5	92.4	99.8	99.5
Yin-Yang	1.85	100	100	98.7	100	100

Supplementary Table 5: Selected codec parameters for codec “DNA-Aeon”.

Parameter	Default	In-silico studies			In-vitro pool experiment		
		Low	*Medium*	*High*	*Medium*	*High*	*Max*
Homopolymer	4	4	4	4	4	4	4
GC-content	0.4-0.6	0.0-1.0	0.0-1.0	0.0-1.0	0.0-1.0	0.0-1.0	0.0-1.0
Package redundancy	0.45	1.68	0.34	0.031	0.32	0.028	0.0
Chunk size	14	25	25	28	20	24	28
Sync value	4	4	4	8	4	12	0
Error correction	CRC	CRC	CRC	CRC	CRC	CRC	nocode
Codeword length	10	10	10	10	10	10	10
CRC threshold	3	3	3	3	3	3	3
Loop	1	1	1	1	1	1	1
Finish	0	0	0	0	0	0	0
Penalty (CRC)	0.1	0.1	0.1	0.1	0.1	0.1	0.1
Penalty (No-Hit)	8	8	8	8	8	8	8

Supplementary Table 6: Selected codec parameters for codec “DNA Fountain”.

Parameter	Default	In-silico studies			In-vitro pool experiment		
		Low	*Medium*	*High*	*Medium*	*High*	*Max*
Alpha	0.07	2.35	0.68	0.19	0.6	0.14	0.0
Payload	32	32	32	34	24	26	27
RS length	-	2	2	0	2	1	0
Hamming distance	100	100	100	100	100	100	100
GC-content	-	0.0-1.0	0.0-1.0	0.0-1.0	0.0-1.0	0.0-1.0	0.0-1.0
Homopolymer	4	4	4	4	4	4	4
Delta	0.05	0.1	0.1	0.1	0.1	0.1	0.1
C-Dist	0.1	0.025	0.025	0.025	0.025	0.025	0.025
Header size	4	4	4	4	4	4	4

Supplementary Table 7: Selected codec parameters for codec “DNA-RS”. As the number of sequences is dependent on the file size s, it is parameterized according to the equations below the table.

Parameter	Default	In-silico studies			In-vitro pool experiment		
		Low	*Medium*	*High*	*Medium*	*High*	*Max*
Mi	6	6	6	6	6	6	6
Mo	14	14	14	14	14	14	14
Index	24	24	24	24	24	24	24
Number of seqs.	-	$f_1(s)$	$f_2(s)$	$f_3(s)$	1110	823	928
Sequence length	-	150	144	144	126	126	102
Inner red. symbols	-	4	2	2	3	3	2

$$f_1(s) = \left\lfloor \frac{875}{8192} s \right\rfloor, \qquad f_2(s) = \left\lfloor \frac{855}{15360} s \right\rfloor, \qquad f_3(s) = \left\lfloor \frac{570}{15360} s \right\rfloor$$

Supplementary Table 8: Selected codec parameters for codec "HEDGES". As the sequence length is dependent on the file size s and the number of packets, it is parameterized according to the equations below the table.

Parameter	Default	In-silico studies		In-vitro pool experiment
		Low	*Medium*	
Code rate index	-	3	1	1
Sequence length	-	$f_1(s)$	$f_2(s)$	110
Homopolymer	4	4	4	4
GC window	4-8	4-8	4-8	4-8

$$f_1(s) = 28.0\,s - 1015, \qquad f_2(s) = 44.6\,s - 1338$$

Supplementary Table 9: Selected codec parameters for codec "Yin-Yang".

Parameter	Default	In-silico studies	In-vitro pool experiment
Homopolymer	4	4	4
GC-content	0.6	0.75	0.75
Search count	100	100	100
Segment length	120	140	110

Supplementary Table 10: Sequence properties per codec and code rate for the pool experiment.

Codec	File size	Code rate	Count	Ratio	Length	Suffix
	kB	bit nt^{-1}	# seqs.	%	nt	
DNA-Aeon	17	1.01	1150	10.2	120	AGG
	19	1.51	834	7.4	124	ACC
	19	1.81	695	6.2	124	AAA
DNA Fountain	17	1.00	1162	10.3	120	CCA
	19	1.47	854	7.6	124	CAC
	19	1.74	722	6.4	124	ATT
DNA-RS	17	1.00	1110	9.8	126	GAG
	19	1.50	823	7.3	126	CTG
	19	1.64	928	8.2	102	CGT
Goldman	5	0.34	1032	9.1	117	TAT
HEDGES	17	0.99	1275	11.3	110	GTC
Yin-Yang	19	1.82	708	6.3	121	TCG

Supplementary Table 11: Comparisons of achieved storage densities. Note that both Organick et al.[2] and Grass et al.[11,38] report slightly different storage densities in their respective studies (i.e., 17 EB g^{-1} by Organick et al.[2]). This is due to different assumptions for calculation, see Supplementary Note 2. In this table, all calculations were harmonized to facilitate fair comparisons. Synthesis providers are abbreviated to TW (Twist Biosciences) and GS (Genscript).

**Codec**	**DNA-Aeon**		**DNA-RS**		**Literature**	
**Scenario**	**High-F.**	**Low-F.**	**High-F.**	**Low-F.**	**Organick et al.[2]**	**Grass et al.[11,38]**
File size / bit	139 264		139 264		255 512[a]	663 168
Sequence count	1 150		1 110		2 042	4 991
Min. phys. red.	2.0	6.6	2.0	6.6	10	3898
Sequencing depth	30		30		35	372
State	double-stranded		double-stranded		single-stranded	double-stranded
Synthesis provider	TW	GS	TW	GS	TW	GS
Considering only payload						
Length / nt	120		126		110	117
Code rate / bit nt^{-1}	1.01		1.00		1.14	1.14
Stor. dens. / EB g^{-1}	57.4	17.4	56.9	17.2	25.9	0.033
Considering payload and primer adapters						
Length / nt	161		167		150	158
Code rate / bit nt^{-1}	0.75		0.76		0.83	0.84
Stor. dens. / EB g^{-1}	42.6	13.0	43.2	12.9	19.0	0.025
Considering all nucleotides, including suffix and padding						
Length / nt	170		170		150	158
Code rate / bit nt^{-1}	0.71		0.74		0.83	0.84
Stor. dens. / EB g^{-1}	40.4	12.3	42.1	12.7	19.0	0.025

[a] The exact file size was extracted from the SI in Ref. [17] using the random-access primers described by both studies, matching file 10. The file size of 0.1 KB reported in the original study[2] does not match the reported storage density and code rate, and was thus assumed to be erroneous.

Supplementary Table 12: Primer sequences used for amplification, qPCR, and sequencing preparation in this study.

**Name**	**Purpose**	**Sequence**
0F	Amplification, qPCR	ACACGACGCTCTTCCGATCT
0R	Amplification, qPCR	AGACGTGTGCTCTTCCGATCT
2FUF	Sequencing prep.	AATGATACGGCGACCACCGAGATCTACACTCTTTCCCTACACGACGCTCTTCCGATCT
2RIF-GM5	Sequencing prep.	CAAGCAGAAGACGGCATACGAGATCACTGTGTGACTGGAGTTCAGACGTGTGCTCTTCCGATCT
2RIF-GM6	Sequencing prep.	CAAGCAGAAGACGGCATACGAGATATTGGCGTGACTGGAGTTCAGACGTGTGCTCTTCCGATCT
2RIF-GM7	Sequencing prep.	CAAGCAGAAGACGGCATACGAGATGATCTGGTGACTGGAGTTCAGACGTGTGCTCTTCCGATCT
2RIF-GM8	Sequencing prep.	CAAGCAGAAGACGGCATACGAGATTCAAGTGTGACTGGAGTTCAGACGTGTGCTCTTCCGATCT
2RIF-GM11	Sequencing prep.	CAAGCAGAAGACGGCATACGAGATGTAGCCGTGACTGGAGTTCAGACGTGTGCTCTTCCGATCT

Supplementary Table 13: qPCR results of experiments in the worst-case scenario. Coverage is calculated based on the calibration curve obtained by serial dilution, shown in Supplementary Figure 18. A standard from the calibration curve was used to standardize the experimentally measured cycle threshold. Each sample was measured in duplicate.

Sample	qPCR Cq	qPCR Cq_{std}	Calc. coverage	Mean coverage
Standard	17.26	17.435	128	128
	17.28	17.455	127	
Cov. 5	21.85	22.025	6.60	6.56
	21.87	22.045	6.51	
Cov. 10	20.85	21.025	12.6	12.5
	20.88	21.055	12.4	
Cov. 25	19.67	19.845	27.1	26.9
	19.69	19.865	26.7	
Cov. 50	18.62	18.795	53.4	52.0
	18.70	18.875	50.7	
Cov. 1000	14.01	14.185	1054	1054
	14.01	14.185	1054	

Supplementary Table 14: qPCR results of experiments in the best-case scenario. Coverage is calculated based on the calibration curve obtained by serial dilution, shown in Supplementary Figure 18. A standard from the calibration curve was used to standardize the experimentally measured cycle threshold. Each sample was measured in duplicate.

Sample	qPCR Cq	qPCR Cq_{std}	Calc. coverage	Mean coverage
Standard	17.09	16.785	137	137
	17.09	16.785	137	
Cov. 2	23.54	23.235	1.99	1.96
	23.58	23.275	1.94	
Cov. 5	22.16	21.855	4.91	4.93
	22.15	21.845	4.95	
Cov. 10	21.20	20.895	9.23	9.23
	21.20	20.895	9.23	
Cov. 25	19.33	19.025	31.5	31.2
	19.36	19.055	30.9	
Cov. 1000	13.70	13.395	1265	1273
	13.68	13.375	1281	

Supplementary Figures

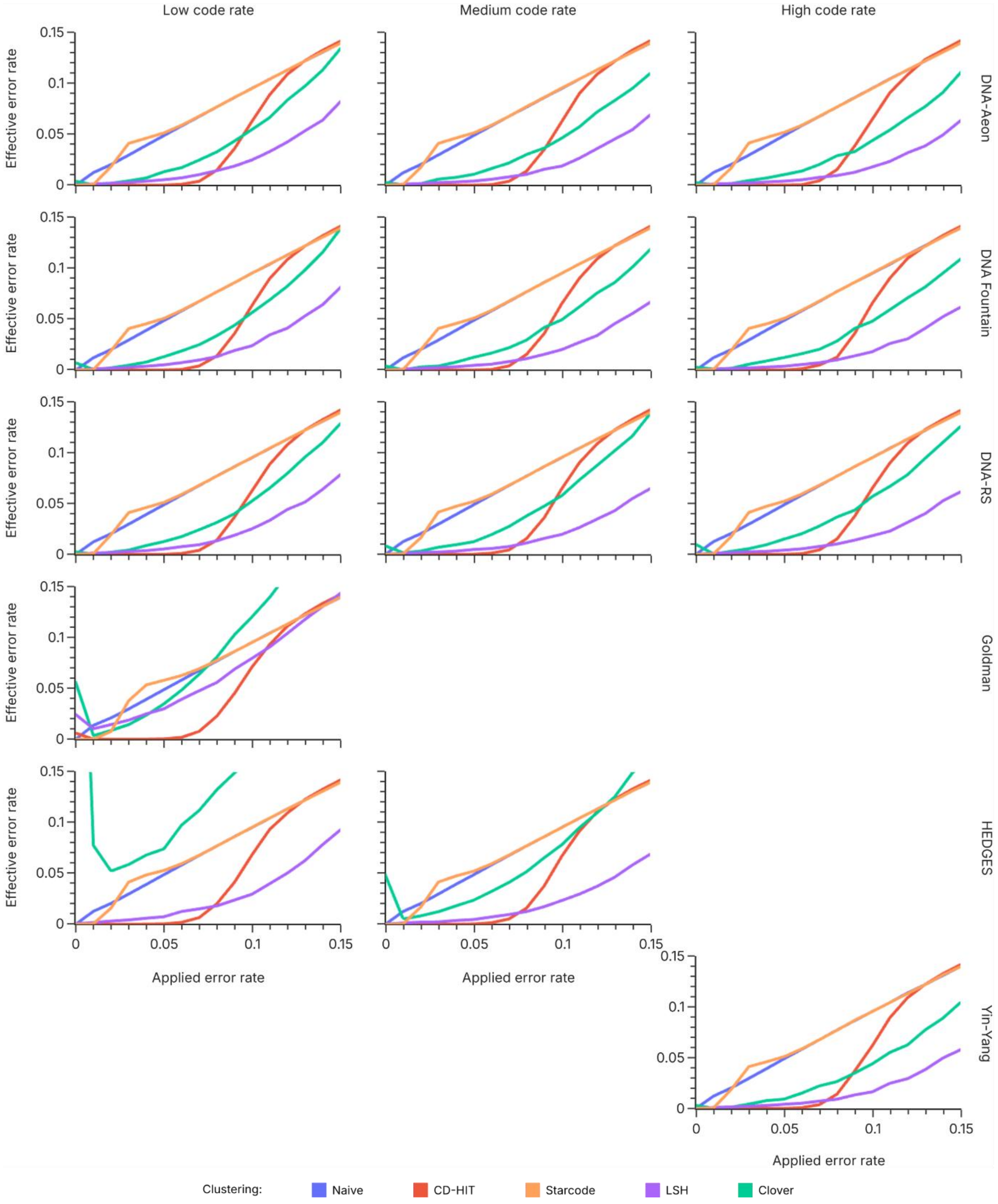

Supplementary Figure 1: Relationship between the effective error rate in clustered reads and the applied error rate during the basic error scenario for each codec-clustering pairing. For each investigated codec (rows), code rate (columns), and clustering algorithm (color), the effective error rate in the clustered reads as determined by mapping to the reference sequences is shown. In all cases, an increase in the error rate in the sequencing reads yields an increase in the error rate in the clustered reads. Source data are provided as a Source Data file.

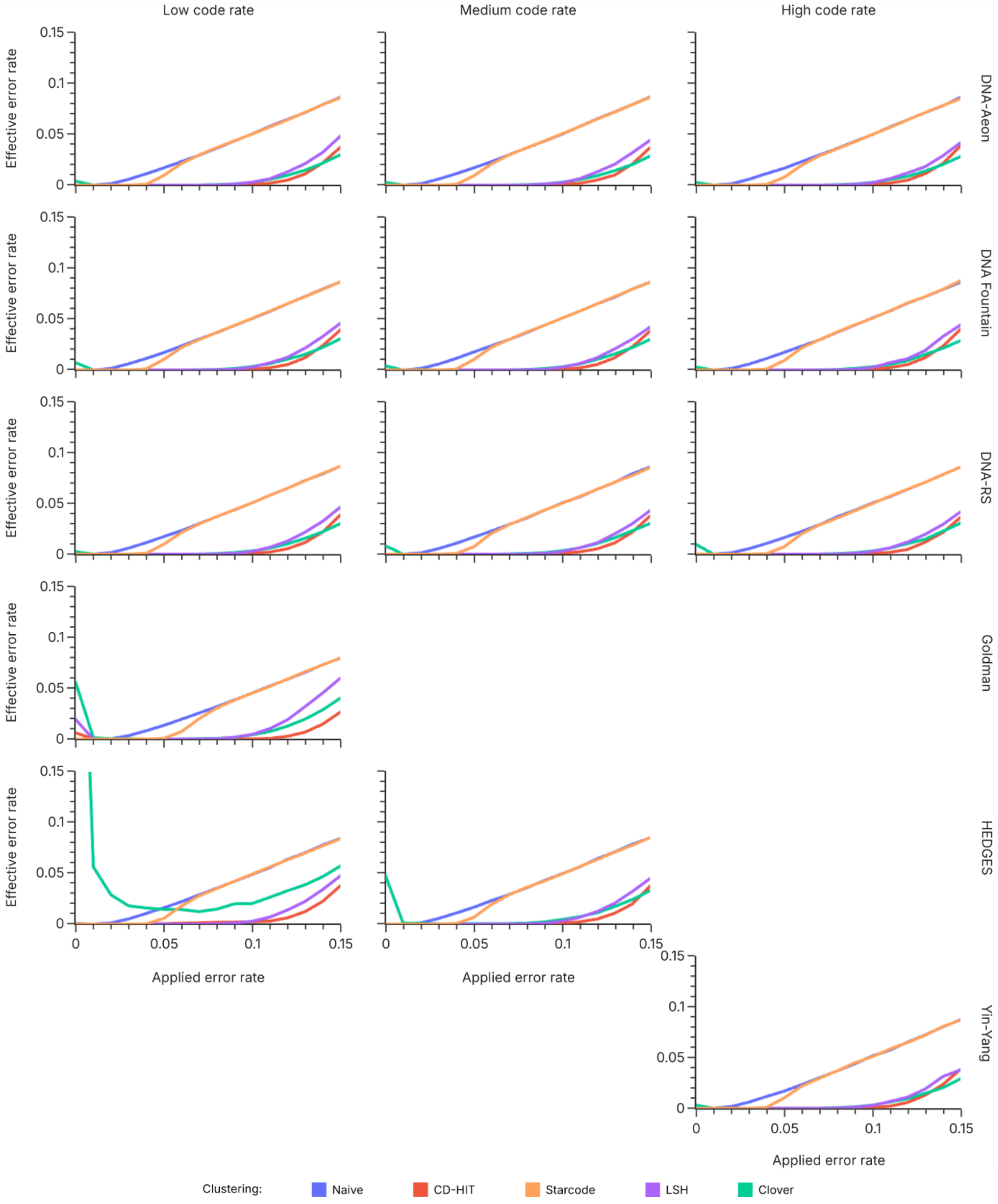

Supplementary Figure 2: Relationship between the effective error rate in the closest clustered reads and the applied error rate during the basic error scenario for each codec-clustering pairing. For each investigated codec (rows), code rate (columns), and clustering algorithm (color), the effective error rate in the clustered reads as determined by mapping to the reference sequences is shown. In contrast to Suppl. Fig. 1, only the closest cluster to each reference sequence is considered. This is most representative of the effective error rate encountered by the decoder. In all cases, an increase in the error rate in the sequencing reads yields an increase in the error rate in the clustered reads. Source data are provided as a Source Data file.

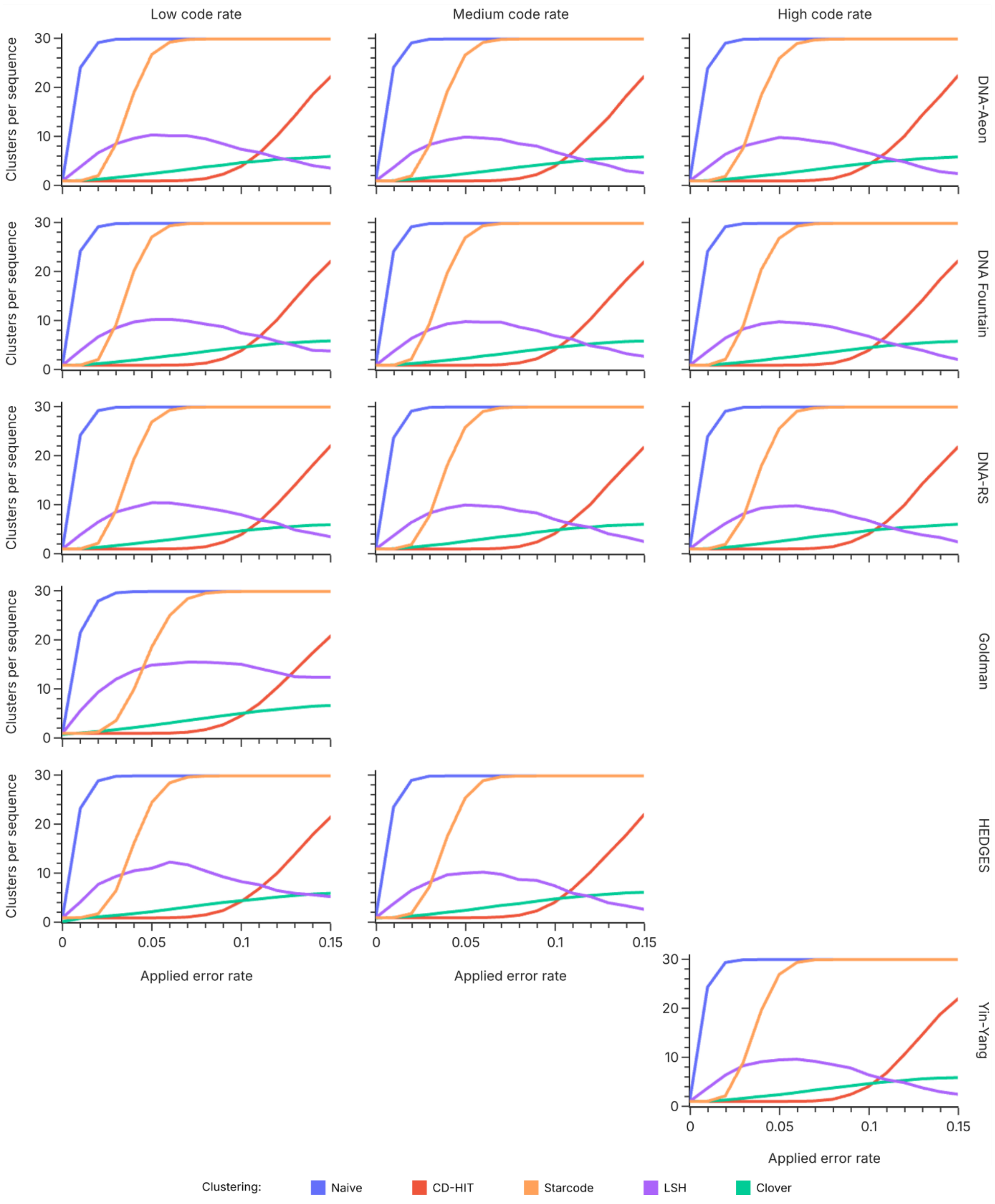

Supplementary Figure 3: Relationship between the number of clusters for each reference seqeunce and the applied error rate during the basic error scenario for each codec-clustering pairing. For each investigated codec (rows), code rate (columns), and clustering algorithm (color), the number of generated clusters for each reference sequence is shown. As the basic error scenario generates exactly 30 erroneous reads per reference sequence, a low number of clusters per sequence is desirable. However, all clustering algorithms generate multiple clusters for each reference sequence as the applied error rate increases. Source data are provided as a Source Data file.

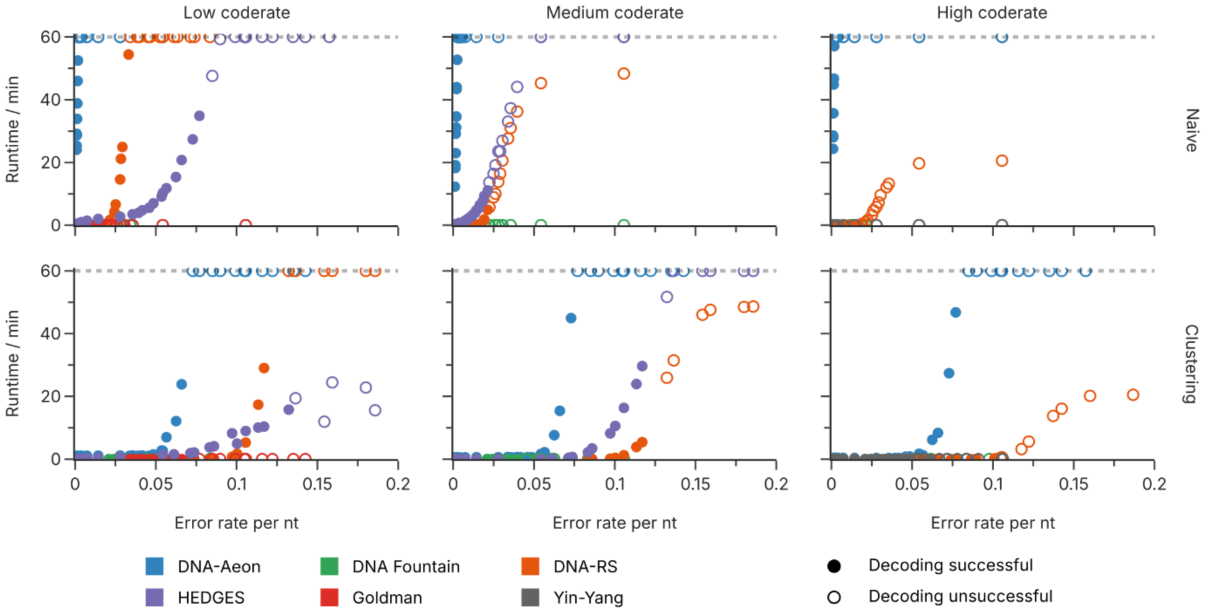

Supplementary Figure 4: Decoding time as a function of error rate in the basic error scenario with combined errors. The runtime of the decoding step is shown for the DNA-Aeon, DNA Fountain, DNA-RS, Goldman, HEDGES, and Yin-Yang codecs at all used code rates, when substitutions, deletions, and insertions are introduced simultaneously at a ratio of 53:45:2. Points correspond to individual runs of the pipeline. Open circles denote individual runs which failed the decoding step, either due to violation of the runtime constraint or due to insufficient error-correction capabilities.

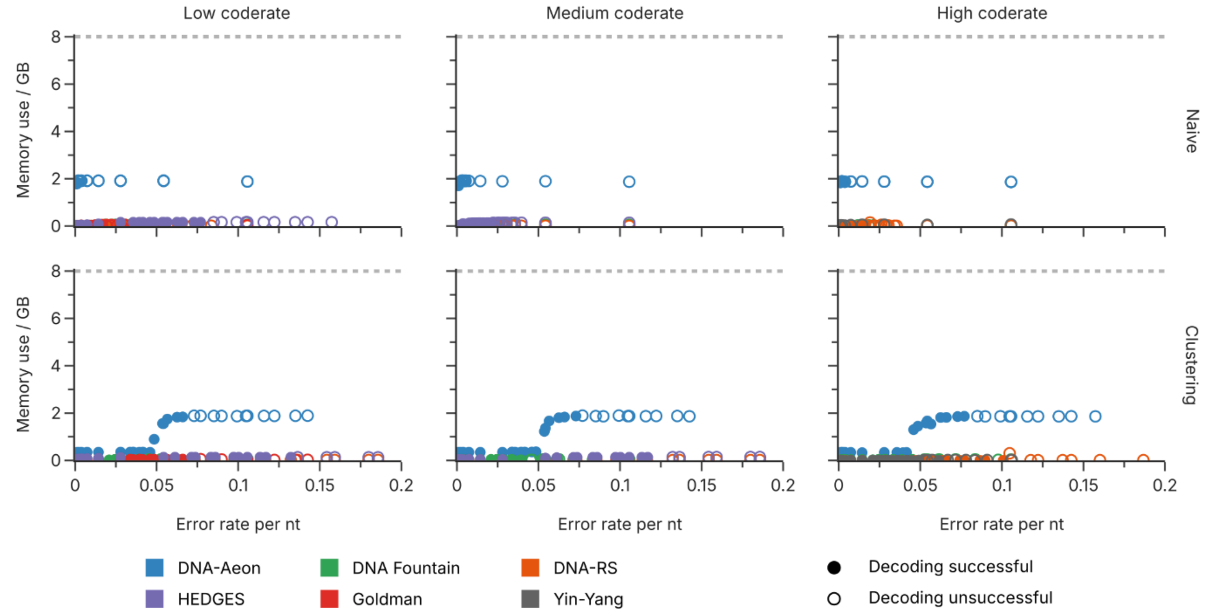

Supplementary Figure 5: Memory use as a function of error rate in the basic error scenario with combined errors. The memory use of the decoding step is shown for the DNA-Aeon, DNA Fountain, DNA-RS, Goldman, HEDGES, and Yin-Yang codecs at all used code rates, when substitutions, deletions, and insertions are introduced simultaneously at a ratio of 53:45:2. Points correspond to individual runs of the pipeline. Open circles denote individual runs which failed the decoding step, either due to violation of the runtime constraint or due to insufficient error-correction capabilities.

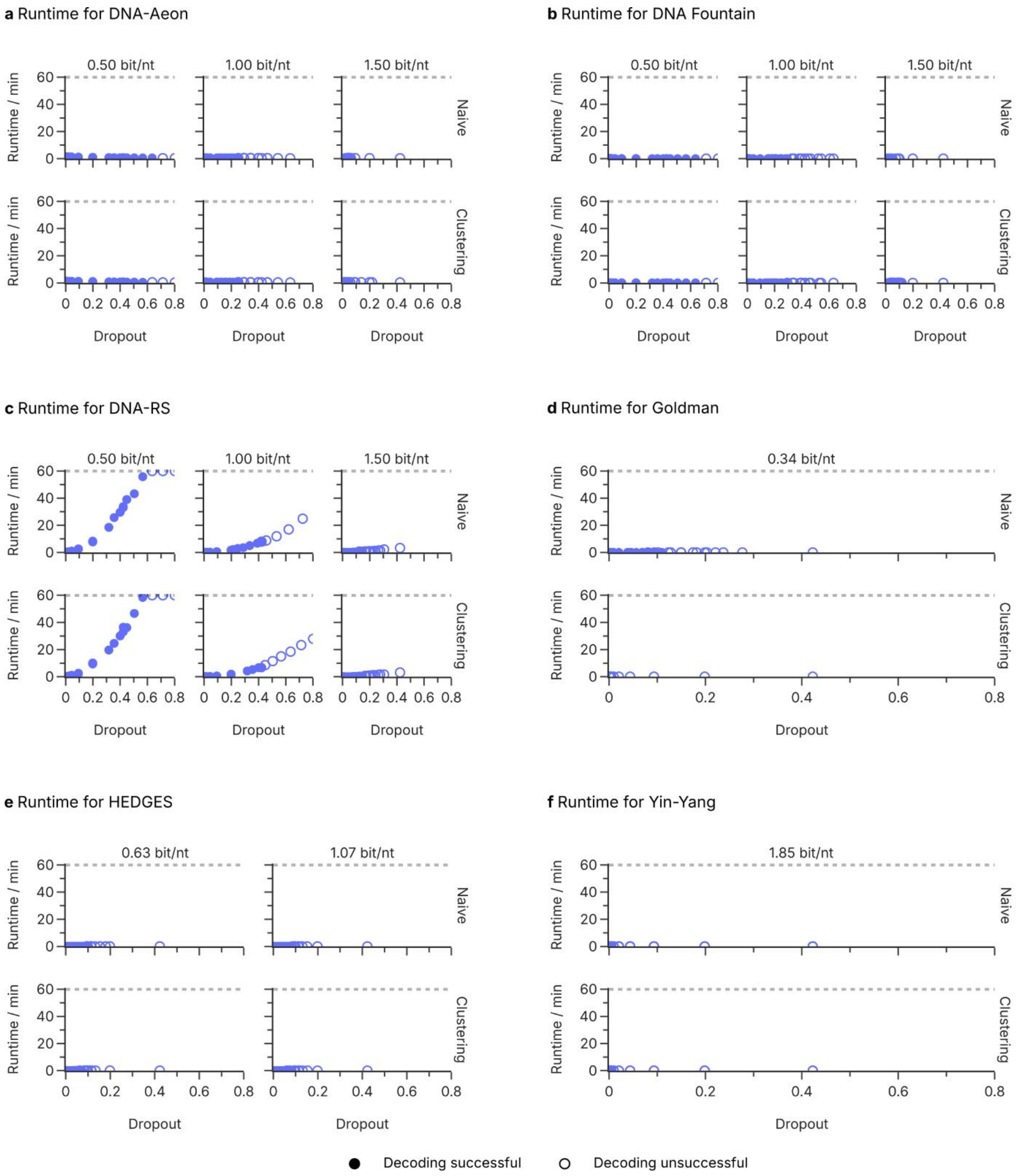

Supplementary Figure 6: Decoding time as a function of sequence dropout in the basic error scenario with sequence loss. The runtime of the decoding step is shown for the DNA-Aeon (**a**), DNA Fountain (**b**), DNA-RS (**c**), Goldman (**d**), HEDGES (**e**), and Yin-Yang (**f**) codecs at all used code rates, when sequence loss is introduced at various rates. Points correspond to individual runs of the pipeline at the specified sequence loss. Open circles denote individual runs which failed the decoding step, either due to violation of the runtime constraint or due to insufficient error-correction capabilities. Source data are provided as a Source Data file.

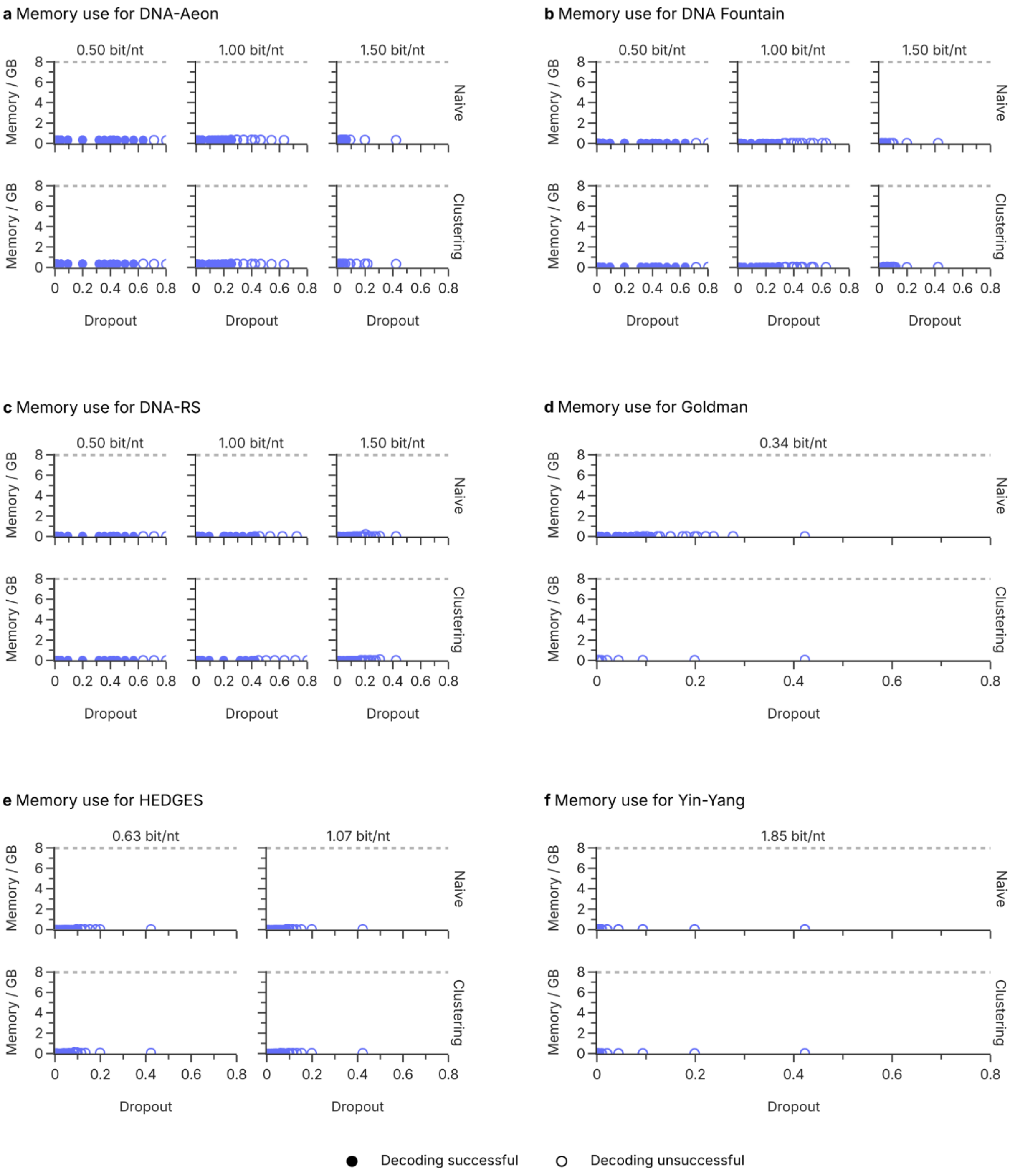

Supplementary Figure 7: Memory use as a function of sequence dropout in the basic error scenario with sequence loss. The memory use of the decoding step is shown for the DNA-Aeon (**a**), DNA Fountain (**b**), DNA-RS (**c**), Goldman (**d**), HEDGES (**e**), and Yin-Yang (**f**) codecs at all used code rates, when sequence loss is introduced at various rates. Points correspond to individual runs of the pipeline at the specified sequence loss. Open circles denote individual runs which failed the decoding step, either due to violation of the runtime constraint or due to insufficient error-correction capabilities. Source data are provided as a Source Data file.

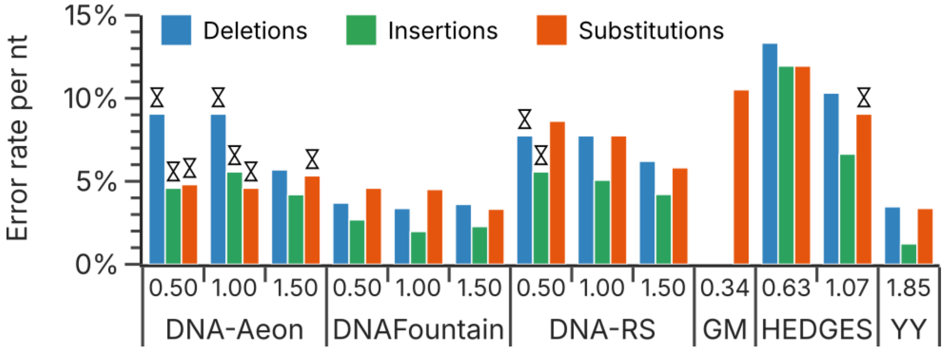

Supplementary Figure 8: Tolerance against individual errors in the basic error scenario. Performance of all codecs in the basic error scenario, introducing only deletions (blue), insertions (green), or substitutions (orange). All codecs use the best-performing clustering algorithm identified in Supplementary Table 3. Conditions indicated with an hourglass (⧖) were limited by the time constraint (see Supplementary Figs. 9 and 10). Source data are provided as a Source Data file.

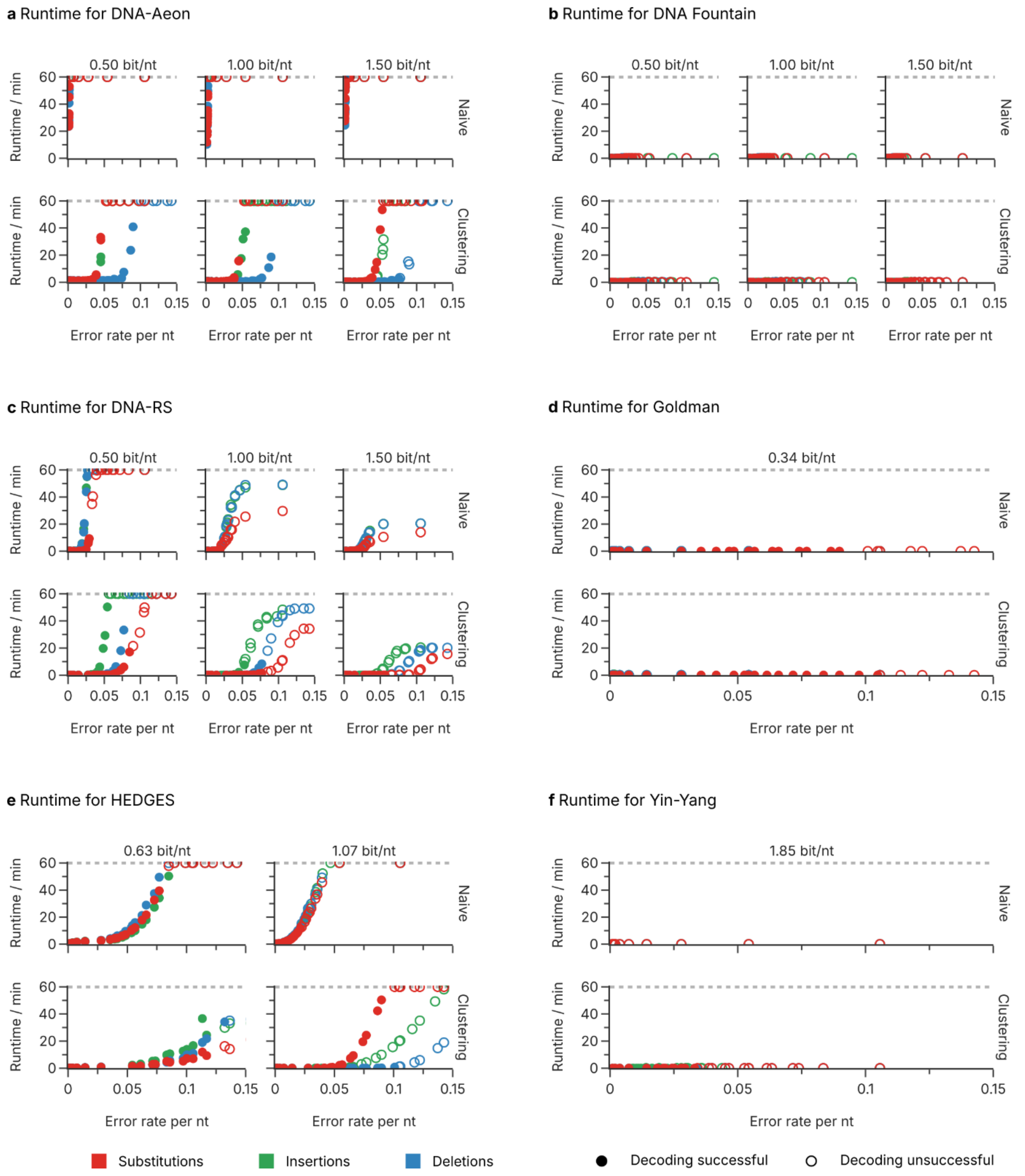

Supplementary Figure 9: Decoding time as a function of error rate in the basic error scenario with individual errors. The runtime of the decoding step is shown for the DNA-Aeon (**a**), DNA Fountain (**b**), DNA-RS (**c**), Goldman (**d**), HEDGES (**e**), and Yin-Yang (**f**) codecs at all used code rates, when substitutions (red), deletions (blue), or insertions (green) are introduced individually. Points correspond to individual runs of the pipeline at the specified error rate and error type. Open circles denote individual runs which failed the decoding step, either due to violation of the runtime constraint or due to insufficient error-correction capabilities. Source data are provided as a Source Data file.

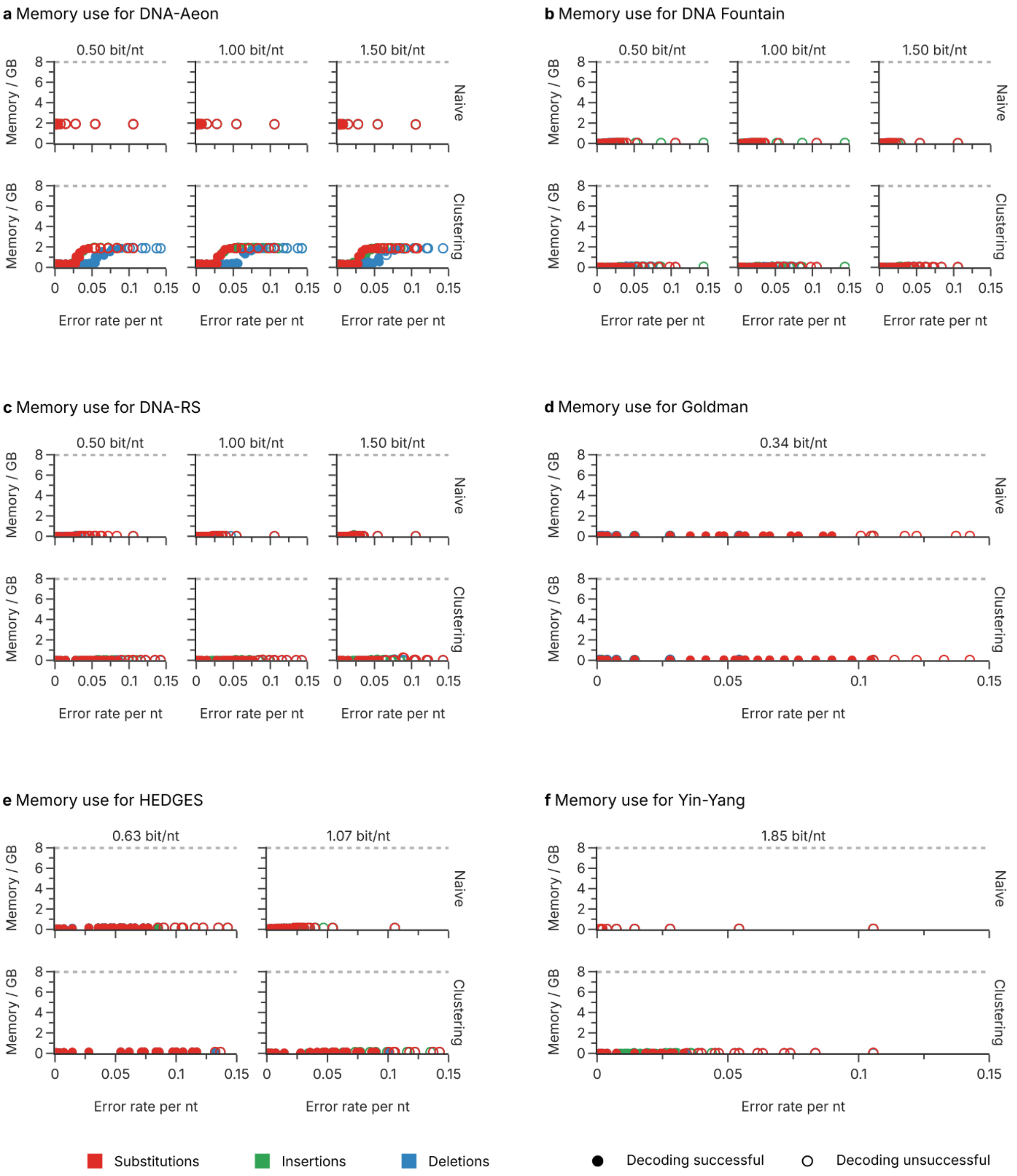

Supplementary Figure 10: Memory use as a function of error rate in the basic error scenario with individual errors. The memory use of the decoding step is shown for the DNA-Aeon (**a**), DNA Fountain (**b**), DNA-RS (**c**), Goldman (**d**), HEDGES (**e**), and Yin-Yang (**f**) codecs at all used code rates, when substitutions (red), deletions (blue), or insertions (green) are introduced individually. Points correspond to individual runs of the pipeline at the specified error rate and error type. Open circles denote individual runs which failed the decoding step, either due to violation of the runtime constraint or due to insufficient error-correction capabilities. Source data are provided as a Source Data file.

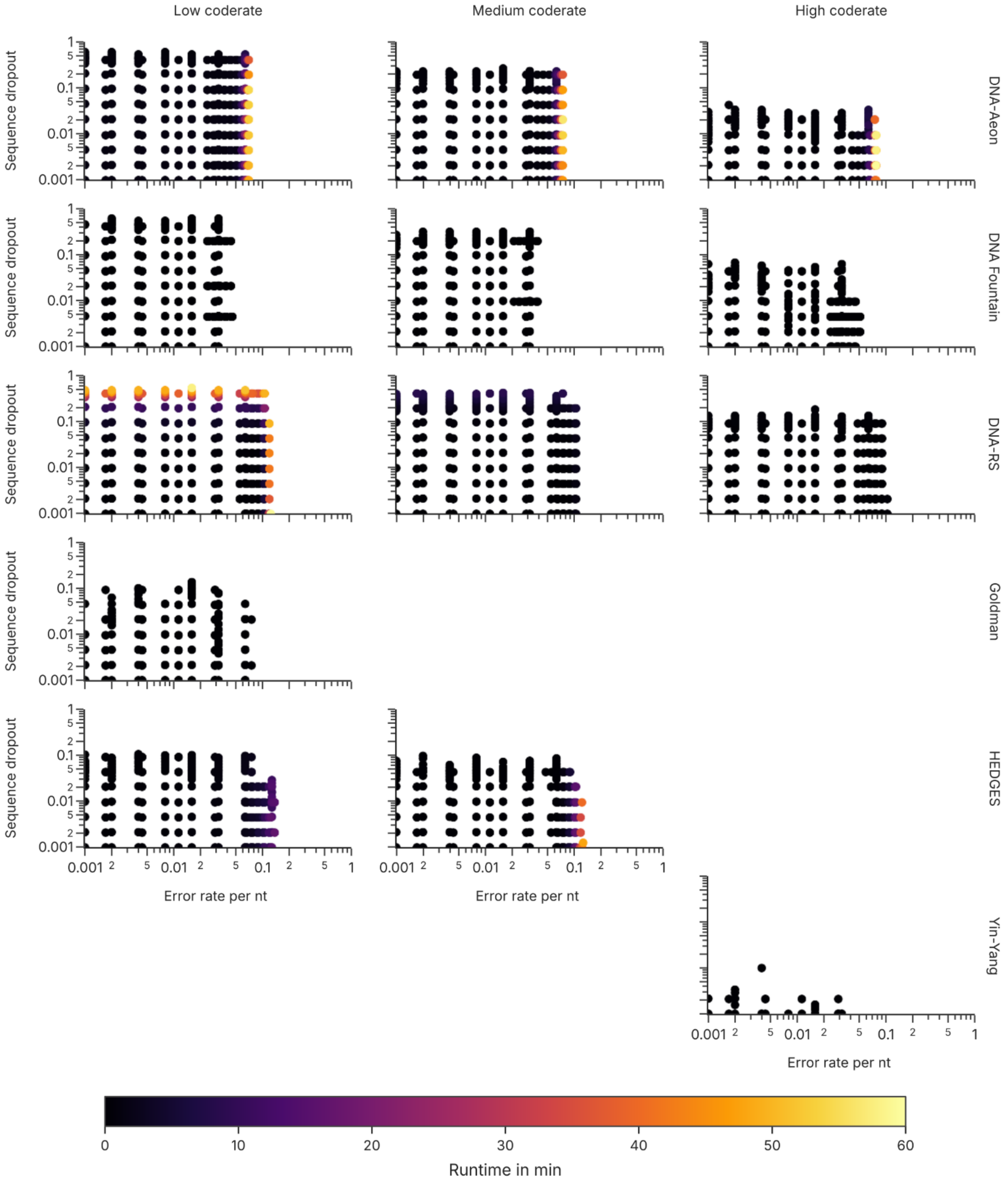

Supplementary Figure 11: Decoding time as a function of overall error rate and sequence dropout in the two-parameter sensitivity analysis. The runtime of the decoding step is shown for the DNA-Aeon, DNA Fountain, DNA-RS, Goldman, HEDGES, and Yin-Yang codecs (top to bottom), at the different code rates (left to right). Points correspond to individual runs of the pipeline at the specified error rate and sequence dropout. Only individual runs which led to successful decoding are shown (i.e., runs which violated the runtime constraint or failed due to insufficient error-correction capabilities are not shown). Source data are provided as a Source Data file.

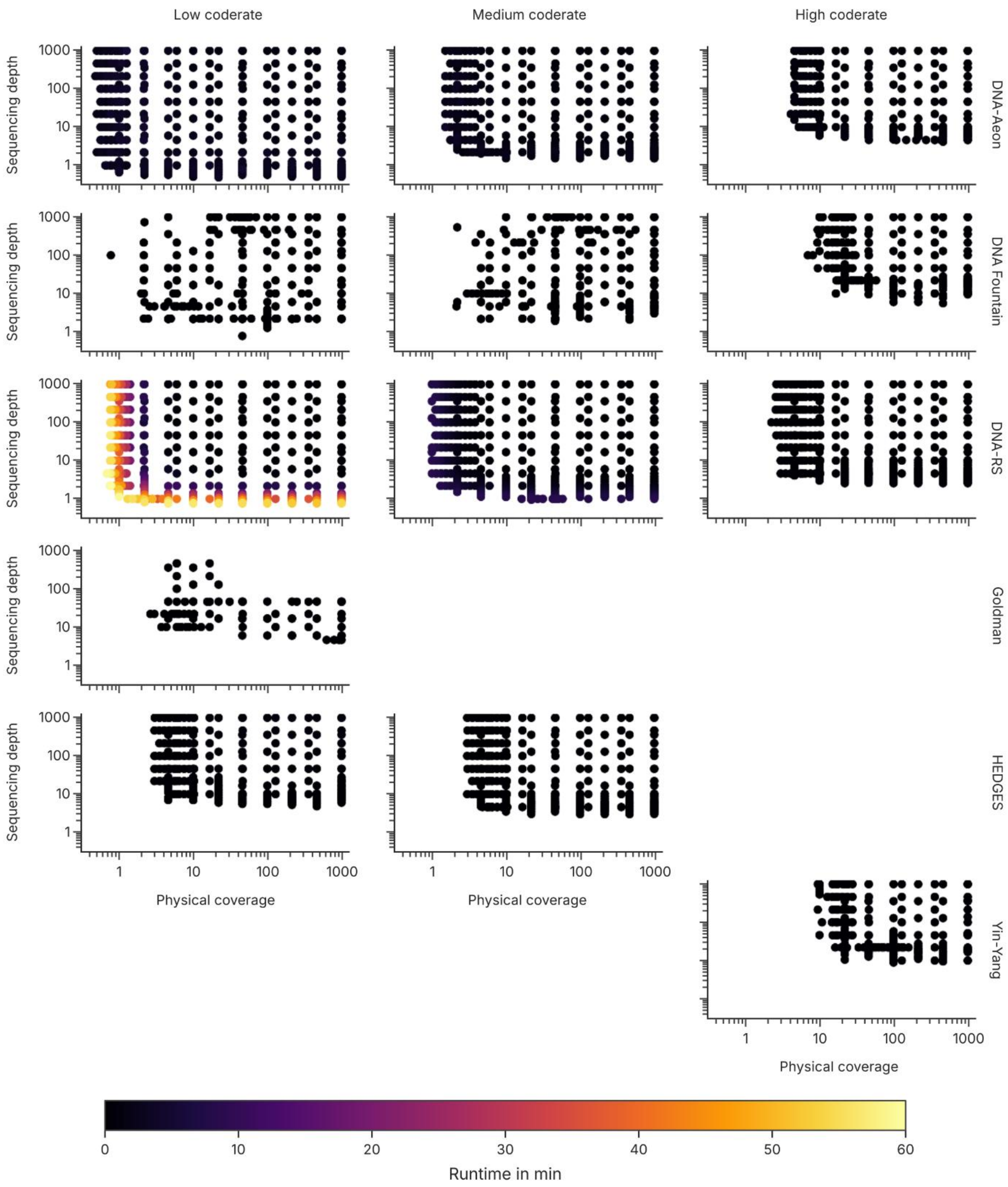

Supplementary Figure 12: Decoding time as a function of sequencing depth and physical coverage in the high-fidelity scenario. The runtime of the decoding step is shown for the DNA-Aeon, DNA Fountain, DNA-RS, Goldman, HEDGES, and Yin-Yang codecs (top to bottom), at the different code rates (left to right). Points correspond to individual runs of the pipeline at the specified physical coverage and sequencing depth of the best-case scenario. Only individual runs which led to successful decoding are shown (i.e., runs which violated the runtime constraint or failed due to insufficient error-correction capabilities are not shown). Source data are provided as a Source Data file.

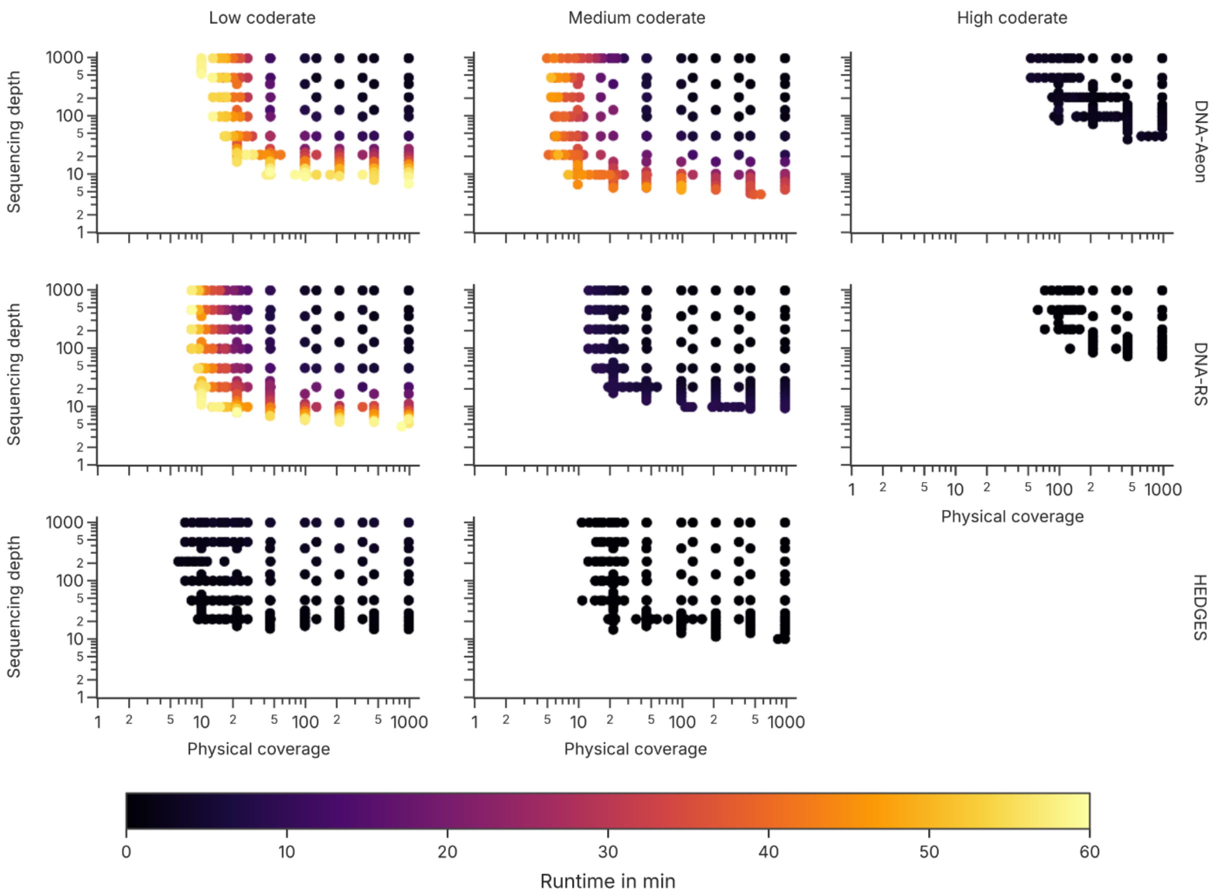

Supplementary Figure 13: Decoding time as a function of sequencing depth and physical coverage in the low-fidelity scenario. The runtime of the decoding step is shown for the DNA-Aeon, DNA-RS, and HEDGES codecs (top to bottom, other codecs failed to successfully decode at all), at the different code rates (left to right). Points correspond to individual runs of the pipeline at the specified physical coverage and sequencing depth of the worst-case scenario. Only individual runs which led to successful decoding are shown (i.e., runs which violated the runtime constraint or failed due to insufficient error-correction capabilities are not shown). Source data are provided as a Source Data file.

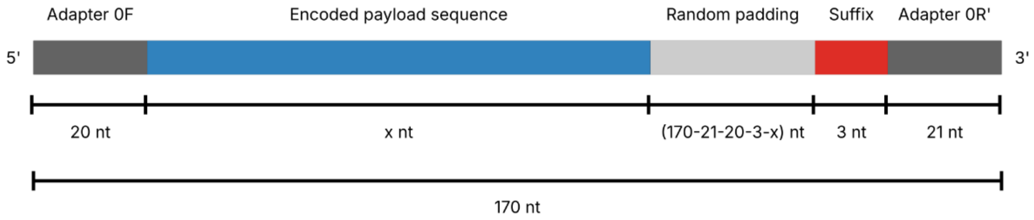

Supplementary Figure 14: Sequence design for pool experiments. The sequences are composed of the forward and reverse adapter for amplification (dark gray), the encoded payload sequence as generated by a codec (blue), a random padding to pad the combined sequence to 170 nt (light gray), and a short suffix for identification (red). See Supplementary Table 10 or additional information on sequence properties of the individual codecs.

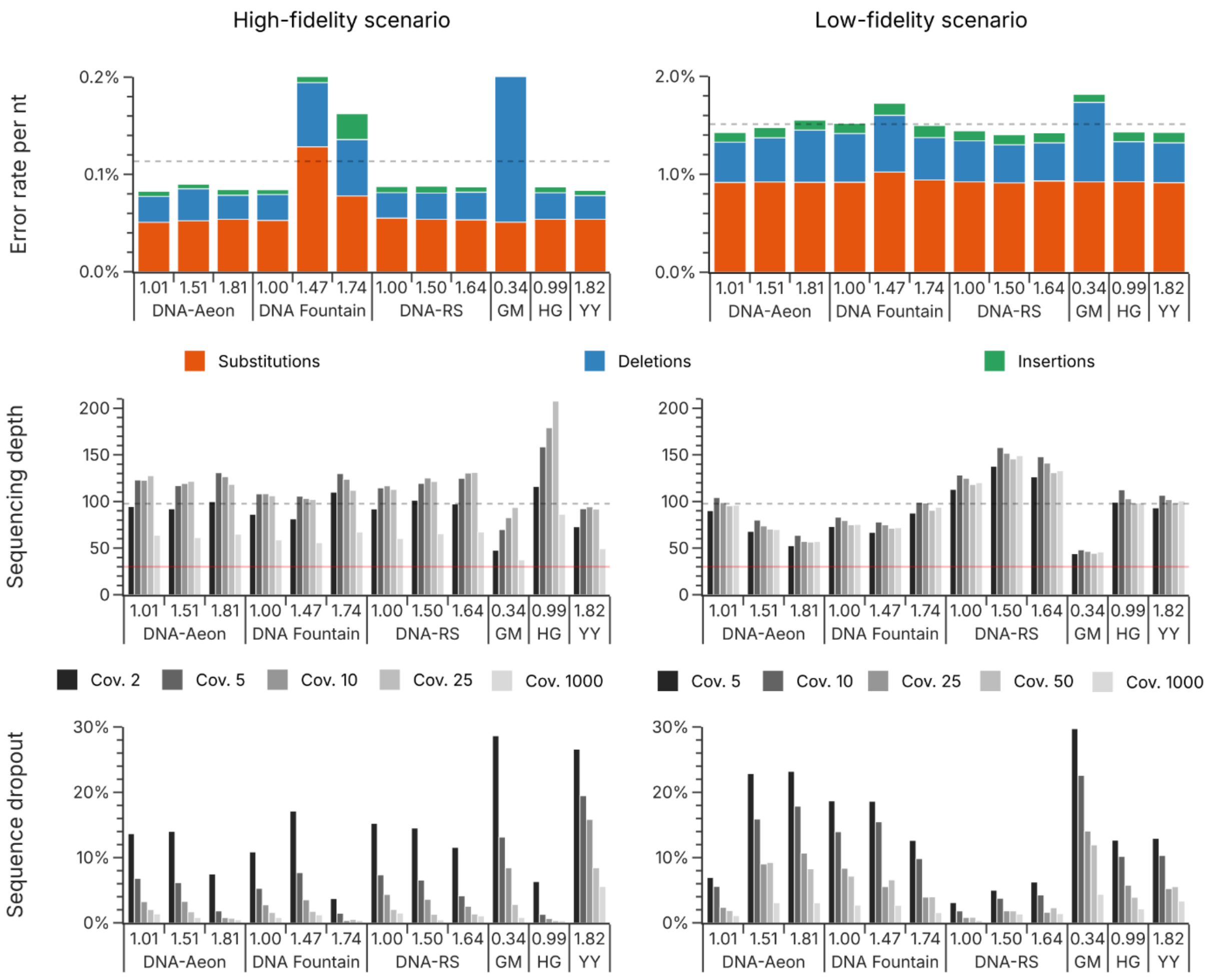

Supplementary Figure 15: Comparison of the mean error rates, sequencing depths, and rates of sequence dropout between codecs and scenarios in the pool experiments. The error rates (top row) in the high- (left) and low-fidelity scenario (right) are composed of substitutions (orange), deletions (blue), and insertions (green). In each case, the sequencing data from the experiment with a coverage of 1000x was used for error analysis. The dotted line represents the mean across all codecs. The sequencing depth highlights differences between codecs (groups) and coverages (individual colors). The mean sequencing depth (middle row) across all codecs is shown with a dotted line. The solid red line indicates a sequencing depth of 30x, to which all sequencing data was downsampled for the decoding experiments, as well as the analysis of sequence dropout. The sequence dropout (bottom row) shows differences between codecs (groups) and coverages (individual colors). To quantify the sequence dropout, the full sequencing data was downsampled to a sequencing depth of 30x ten times, and the average fraction of sequences without a corresponding read are reported. Source data are provided as a Source Data file.

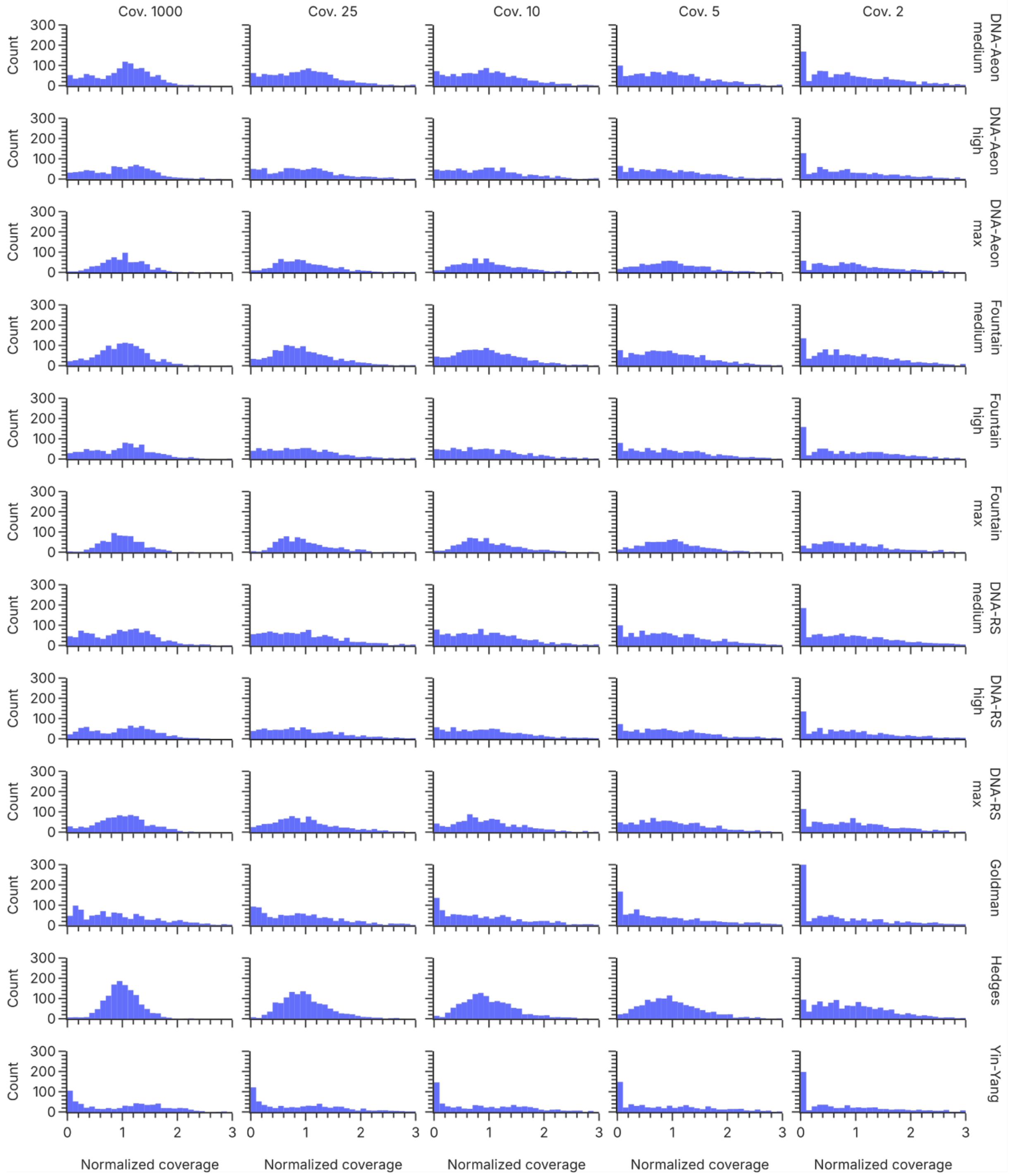

Supplementary Figure 16: Coverage distributions between codecs and coverages in the pool experiment using the high-fidelity scenario. The histograms show the homogeneity of the sequence coverage, normalized to the mean coverage. The more skewed the coverage distribution, the less homogeneous the representation of each sequence in the oligo pool. Source data are provided as a Source Data file.

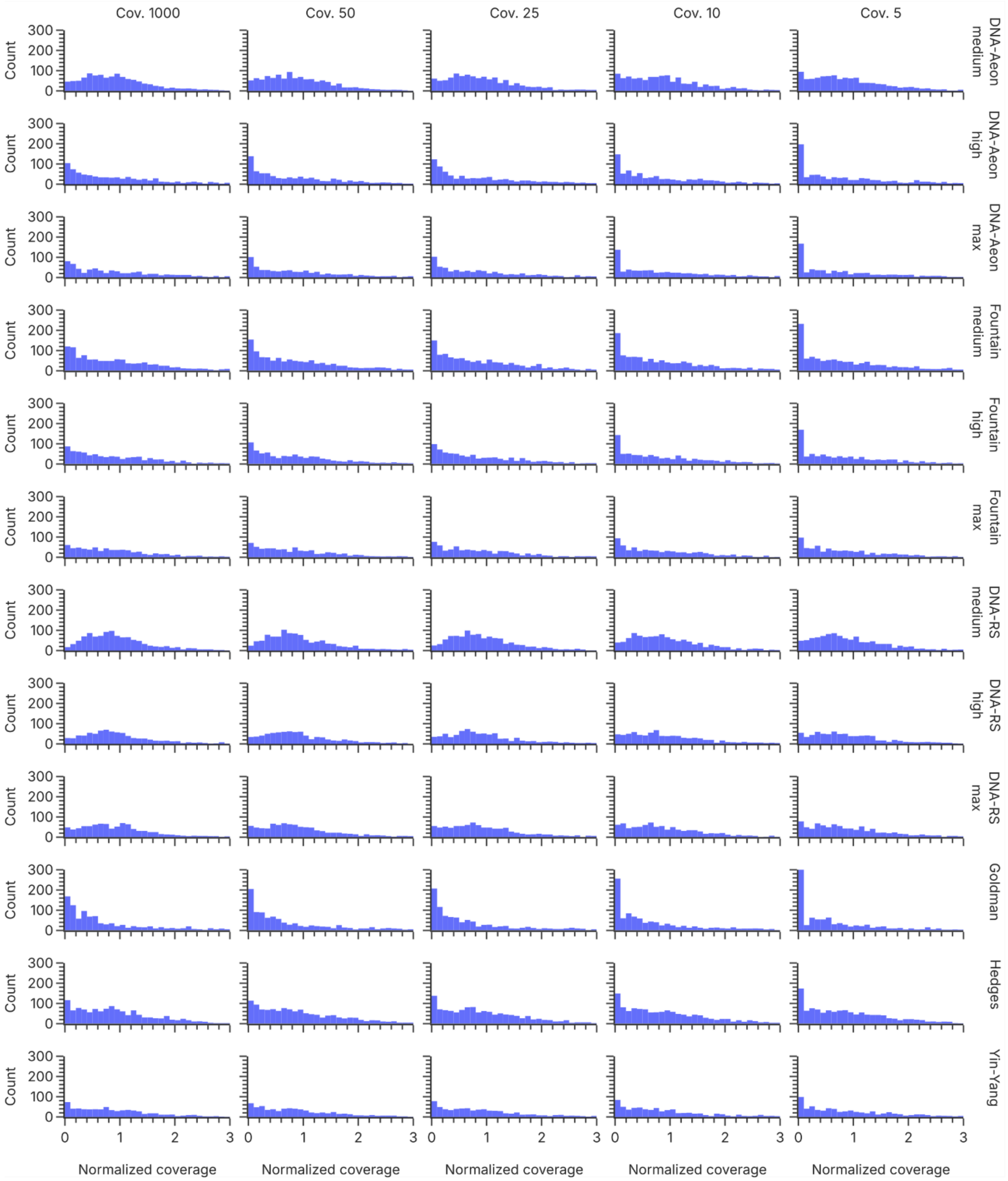

Supplementary Figure 17: Coverage distributions between codecs and coverages in the pool experiment using the low-fidelity scenario. The histograms show the homogeneity of the sequence coverage, normalized to the mean coverage. The more skewed the coverage distribution, the less homogeneous the representation of each sequence in the oligo pool. Source data are provided as a Source Data file.

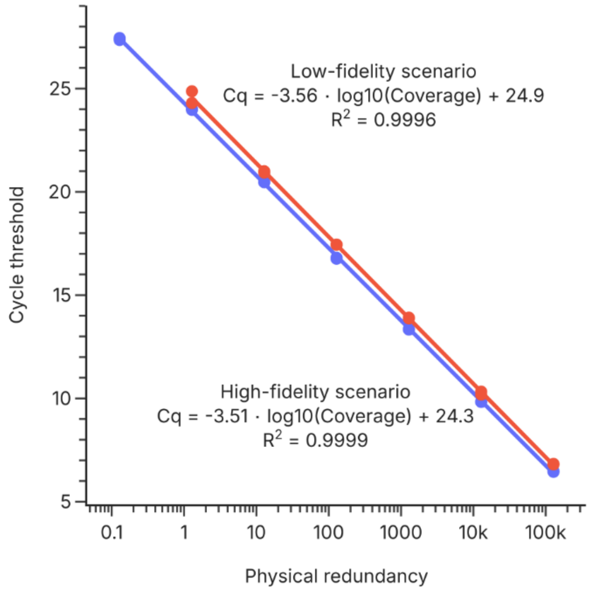

Supplementary Figure 18: qPCR calibration curves in the high- and low-fidelity scenarios. The calibration curves were generated by serial dilutions of the two master pools generated from the first amplification PCR after synthesis. Conversion of mass concentration to coverage was performed assuming a physical coverage of 509074x per ng, and a sample volume of 5 µL (see Methods). Source data are provided as a Source Data file.

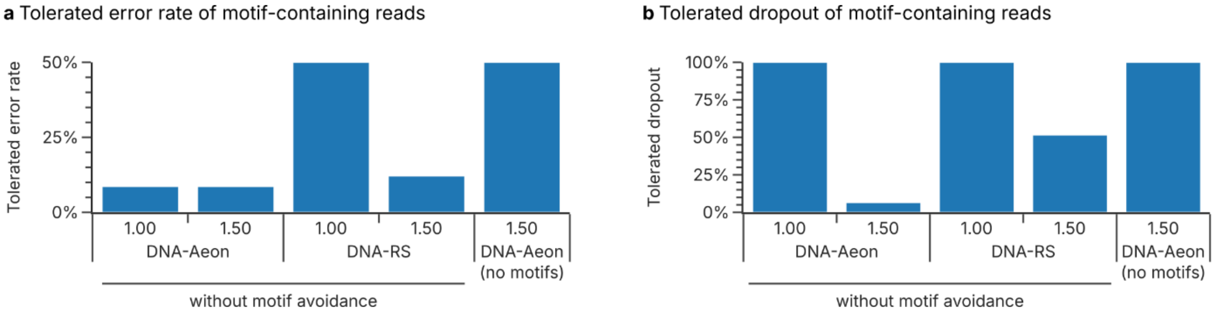

Supplementary Figure 19: Benefits of motif avoidance during error-correction coding. The DNA-Aeon codec supports custom codebooks to avoid the introduction of user-defined motifs into the design sequences. To test the potential benefit of motif avoidance, a baseline scenario of 3% errors (53% substitutions, 45% deletions, 2% insertions) and 1% sequence dropout was modified such that all sequences containing any of the 46 motifs with biological meaning defined in the MESA DNA Simulator by Schwarz et al.[39] either had drastically increased error rates (**a**) or were drastically more likely to drop out of the sequencing data (**b**). Using a custom codebook avoiding these motifs with DNA-Aeon (right-most bar) precluded any negative effect of increased error rates or sequence loss, as expected. Source data are provided as a Source Data file.

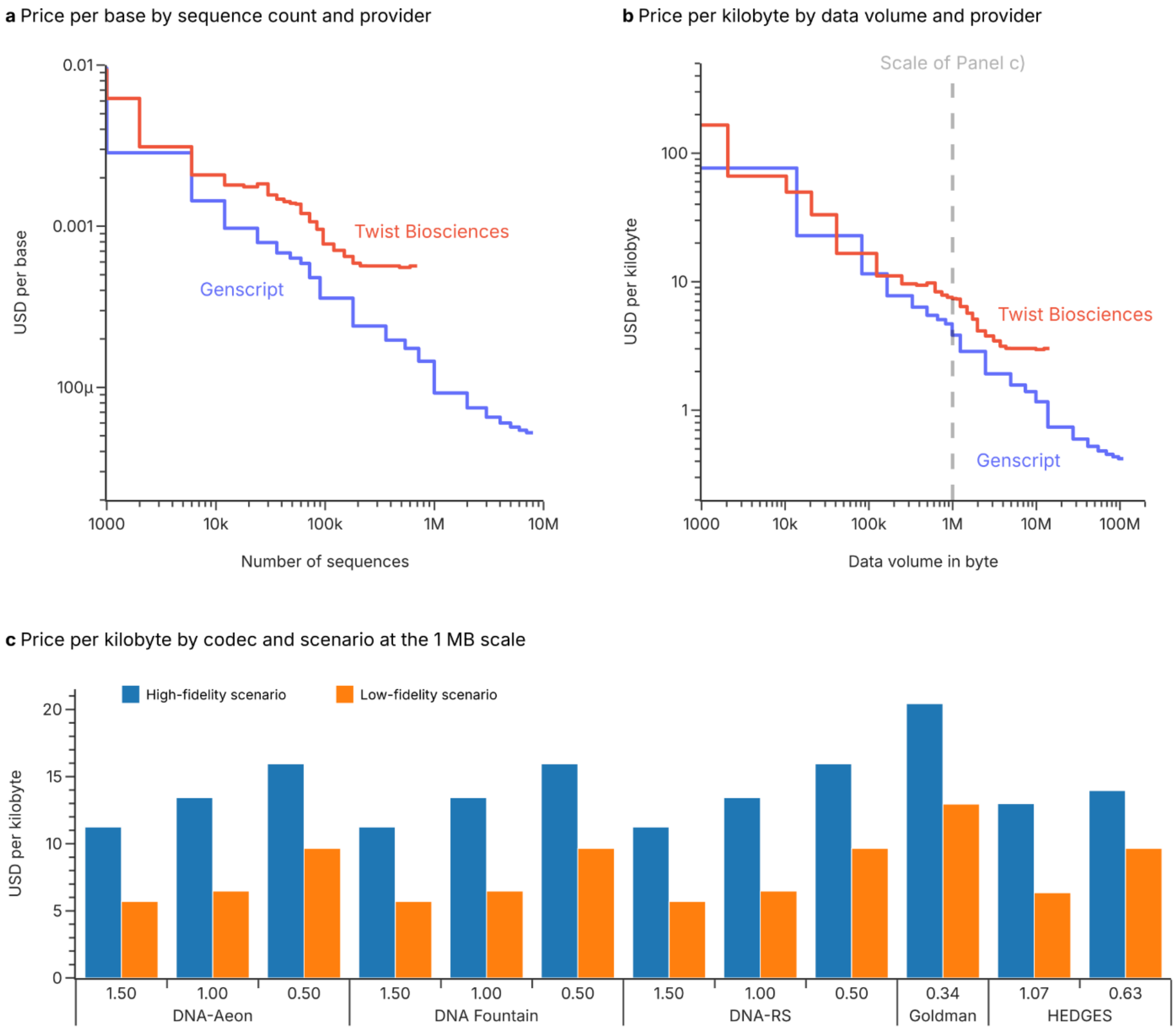

Supplementary Figure 20: Cost comparison of codecs by synthesis provider and scenario. (**a**) The cost structure of the two commercial providers analysed in this study depends strongly on the number of synthesized sequences (assuming a fixed sequence length of 150 nt). (**b**) Using a fixed code rate of 1.5 bit/nt for synthesis by Twist Biosciences and 1.0 bit/nt for Genscript yields comparable prices per kilobyte by data volume. (**c**) Comparing the costs for synthesis across all codecs and code rates tested in this study, in both the high- (blue) and low-fidelity scenarios (orange) and at a fixed data volume of one megabyte, reveals only minor differences. However, the costs for low-fidelity synthesis are about half as high as the high-fidelity synthesis across most codecs. Source data are provided as a Source Data file.

Supplementary References

1. Press, W. H. whpress/HEDGES: HEDGES Error Correcting Code for Engineered DNA, Fixes Error Substitutions, Deletions, and Insertions. GitHub (2025).

2. Organick, L. *et al.* Probing the physical limits of reliable DNA data retrieval. *Nat. Commun.* **11**, 1–7 (2020).

3. Chen, Y.-J. *et al.* Quantifying molecular bias in DNA data storage. *Nat. Commun.* **11**, 1–9 (2020).

4. Gimpel, A. L. *et al.* Deep learning uncovers sequence-specific amplification bias in multi-template PCR. bioRxiv, 2024.09.20.614030. Preprint at https://doi.org/10.1101/2024.09.20.614030 (2024).

5. Gimpel, A. L., Stark, W. J., Heckel, R. & Grass, R. N. A digital twin for DNA data storage based on comprehensive quantification of errors and biases. *Nat. Commun.* **14**, 6026 (2023).

6. Qiao, H. *et al.* Oligo replication advantage driven by GC content and Gibbs free energy. *Biotechnol. Lett.* **44**, 1189–1199 (2022).

7. Heinis, T., Sokolovskii, R. & Alnasir, J. J. Survey of Information Encoding Techniques for DNA. *ACM Comput. Surv.* **56**, 1–30 (2024).

8. Ceze, L., Nivala, J. & Strauss, K. Molecular digital data storage using DNA. *Nat. Rev. Genet. 2019 208* **20**, 456–466 (2019).

9. Church, G. M., Gao, Y. & Kosuri, S. Next-generation digital information storage in DNA. *Science* **337**, 1628 (2012).

10. Goldman, N. *et al.* Towards practical, high-capacity, low-maintenance information storage in synthesized DNA. *Nature* **494**, 77–80 (2013).

11. Grass, R. N., Heckel, R., Puddu, M., Paunescu, D. & Stark, W. J. Robust Chemical Preservation of Digital Information on DNA in Silica with Error-Correcting Codes. *Angew. Chem. Int. Ed.* **54**, 2552–2555 (2015).

12. Tabatabaei Yazdi, S. M. H., Yuan, Y., Ma, J., Zhao, H. & Milenkovic, O. A Rewritable, Random-Access DNA-Based Storage System. *Sci. Rep.* **5**, 1–10 (2015).

13. Bornholt, J. *et al.* A DNA-based archival storage system. in *Proceedings of the Twenty-First International Conference on Architectural Support for Programming Languages and Operating Systems (ASPLOS)* 637–649 (Association for Computing Machinery, 2016).

14. Blawat, M. *et al.* Forward Error Correction for DNA Data Storage. *Procedia Comput. Sci.* **80**, 1011–1022 (2016).

15. Yazdi, S. M. H. T., Gabrys, R. & Milenkovic, O. Portable and Error-Free DNA-Based Data Storage. *Sci. Rep.* **7**, 1–6 (2017).

16. Erlich, Y. & Zielinski, D. DNA Fountain enables a robust and efficient storage architecture. *Science* **355**, 950–954 (2017).

17. Organick, L. *et al.* Random access in large-scale DNA data storage. *Nat. Biotechnol.* **36**, 242–248 (2018).

18. Appuswamy, R. *et al.* OligoArchive: Using DNA in the DBMS storage hierarchy. in *Conference on Innovative Data Systems Research* (CIDR, 2019).

19. Wang, Y. *et al.* High capacity DNA data storage with variable-length Oligonucleotides using repeat accumulate code and hybrid mapping. *J. Biol. Eng.* **13**, 89 (2019).

20. Chandak, S. *et al.* Improved read/write cost tradeoff in DNA-based data storage using LDPC codes. in *2019 57th Annual Allerton Conference on Communication, Control, and Computing* 147–156 (2019).

21. Deng, L. *et al.* Optimized Code Design for Constrained DNA Data Storage With Asymmetric Errors. *IEEE Access* **7**, 84107–84121 (2019).

22. Wang, Y., Noor-A-Rahim, M., Gunawan, E., Guan, Y. L. & Poh, C. L. Construction of Bio-Constrained Code for DNA Data Storage. *IEEE Commun. Lett.* **23**, 963–966 (2019).

23. Anavy, L., Vaknin, I., Atar, O., Amit, R. & Yakhini, Z. Data storage in DNA with fewer synthesis cycles using composite DNA letters. *Nat. Biotechnol.* **37**, 1229–1236 (2019).

24. Choi, Y. *et al.* High information capacity DNA-based data storage with augmented encoding characters using degenerate bases. *Sci. Rep.* **9**, 6582 (2019).

25. Press, W. H., Hawkins, J. A., Jones, S. K., Schaub, J. M. & Finkelstein, I. J. HEDGES error-correcting code for DNA storage corrects indels and allows sequence constraints. *Proc. Natl. Acad. Sci.* **117**, 18489–18496 (2020).

26. Antkowiak, P. L. *et al.* Low cost DNA data storage using photolithographic synthesis and advanced information reconstruction and error correction. *Nat. Commun.* **11**, 5345 (2020).

27. Meiser, L. C. *et al.* Reading and writing digital data in DNA. *Nat. Protoc.* **15**, 86–101 (2019).

28. Lenz, A. *et al.* Concatenated Codes for Recovery From Multiple Reads of DNA Sequences. in *2020 IEEE Information Theory Workshop (ITW)* 1–5 (2021).

29. Dimopoulou, M. & Antonini, M. Image storage in DNA using Vector Quantization. in *2020 28th European Signal Processing Conference (EUSIPCO)* 516–520 (2021).

30. Chen, W. *et al.* An artificial chromosome for data storage. *Natl. Sci. Rev.* **8**, nwab028 (2021).

31. Ping, Z. *et al.* Towards practical and robust DNA-based data archiving using the yin–yang codec system. *Nat. Comput. Sci.* **2**, 234–242 (2022).

32. Song, L. *et al.* Robust data storage in DNA by de Bruijn graph-based de novo strand assembly. *Nat. Commun.* **13**, 1–9 (2022).

33. Pan, C. *et al.* Rewritable two-dimensional DNA-based data storage with machine learning reconstruction. *Nat. Commun.* **13**, 2984 (2022).

34. Welzel, M. *et al.* DNA-Aeon provides flexible arithmetic coding for constraint adherence and error correction in DNA storage. *Nat. Commun.* **14**, 628 (2023).

35. Xie, R. *et al.* Study of the error correction capability of multiple sequence alignment algorithm (MAFFT) in DNA storage. *BMC Bioinformatics* **24**, 111 (2023).

36. Zan, X., Xie, R., Yao, X., Xu, P. & Liu, W. A Robust and Efficient DNA Storage Architecture Based on Modulation Encoding and Decoding. *J. Chem. Inf. Model.* **63**, 3967–3976 (2023).

37. Zhao, X. *et al.* Composite Hedges Nanopores codec system for rapid and portable DNA data readout with high INDEL-Correction. *Nat. Commun.* **15**, 9395 (2024).

38. Heckel, R., Mikutis, G. & Grass, R. N. A Characterization of the DNA Data Storage Channel. *Sci. Rep.* **9**, 1–12 (2019).

39. Schwarz, M. *et al.* MESA: automated assessment of synthetic DNA fragments and simulation of DNA synthesis, storage, sequencing and PCR errors. *Bioinformatics* **36**, 3322–3326 (2020).
