# Supplementary material for: Comparison of state-of-the-art error-correction coding for sequence-based DNA data storage
Source: Nat Commun. 2026 Mar 14;17:3963. doi: 10.1038/s41467-026-70548-3 (PMC13133123; doi:10.1038/s41467-026-70548-3)
Supplement: Supplementary file 2 — Reporting Summary [file 41467_2026_70548_MOESM2_ESM.pdf]

nature portfolio

Corresponding author(s): Robert N. Grass

Last updated by author(s): Jan 25, 2026

Reporting Summary

Nature Portfolio wishes to improve the reproducibility of the work that we publish. This form provides structure for consistency and transparency in reporting. For further information on Nature Portfolio policies, see our Editorial Policies and the Editorial Policy Checklist.

Statistics

For all statistical analyses, confirm that the following items are present in the figure legend, table legend, main text, or Methods section.

n/a	Confirmed	
☐	☒	The exact sample size (*n*) for each experimental group/condition, given as a discrete number and unit of measurement
☒	☐	A statement on whether measurements were taken from distinct samples or whether the same sample was measured repeatedly
☒	☐	The statistical test(s) used AND whether they are one- or two-sided<br>*Only common tests should be described solely by name; describe more complex techniques in the Methods section.*
☒	☐	A description of all covariates tested
☒	☐	A description of any assumptions or corrections, such as tests of normality and adjustment for multiple comparisons
☐	☒	A full description of the statistical parameters including central tendency (e.g. means) or other basic estimates (e.g. regression coefficient) AND variation (e.g. standard deviation) or associated estimates of uncertainty (e.g. confidence intervals)
☒	☐	For null hypothesis testing, the test statistic (e.g. *F*, *t*, *r*) with confidence intervals, effect sizes, degrees of freedom and *P* value noted<br>*Give P values as exact values whenever suitable.*
☒	☐	For Bayesian analysis, information on the choice of priors and Markov chain Monte Carlo settings
☒	☐	For hierarchical and complex designs, identification of the appropriate level for tests and full reporting of outcomes
☒	☐	Estimates of effect sizes (e.g. Cohen's *d*, Pearson's *r*), indicating how they were calculated

Our web collection on statistics for biologists contains articles on many of the points above.

Software and code

Policy information about availability of computer code

Data collection	Data collection used NGmerge (v0.3) for read merging, kalign (v3.4) for sequence alignment, and dt4dds (v1.1.0) for workflow simulation, as well as custom code implemented in Python (v3.10) for creating experimental pipelines. Additionally, codecs and clustering algorithms from the literature were used: DNA-Aeon, DNA Fountain, DNA-RS, HEDGES, Goldman codec, Yin-Yang, MMseqs2, Clover, LSH, Starcode, and CD-HIT. The sources for these algorithms from the published literature, as well as the custom code implemented in Python, have been publicly deposited at https://github.com/fml-ethz/dt4dds-benchmark.
Data analysis	Data analysis used NGmerge (v0.3) and BBMap (v39.01) for read merging and mapping, as well as custom code implemented in Python (v3.10) for data compilation and figure generation. The custom code for data analysis has been publicly deposited at https://github.com/fml-ethz/dt4dds-benchmark_notebooks.

For manuscripts utilizing custom algorithms or software that are central to the research but not yet described in published literature, software must be made available to editors and reviewers. We strongly encourage code deposition in a community repository (e.g. GitHub). See the Nature Portfolio guidelines for submitting code & software for further information.

Data

Policy information about availability of data

All manuscripts must include a data availability statement. This statement should provide the following information, where applicable:

- Accession codes, unique identifiers, or web links for publicly available datasets
- A description of any restrictions on data availability
- For clinical datasets or third party data, please ensure that the statement adheres to our policy

The sequencing data generated in this study has been deposited in the European Nucleotide Archive under accession code PRJEB90546.

Research involving human participants, their data, or biological material

Policy information about studies with human participants or human data. See also policy information about sex, gender (identity/presentation), and sexual orientation and race, ethnicity and racism.

Reporting on sex and gender	Sex and gender were not considered in this study.
Reporting on race, ethnicity, or other socially relevant groupings	Race, ethnicity, or other socially relevant groupings were not considered in this study.
Population characteristics	Population characteristics were not considered in this study.
Recruitment	No participants were recruited for this study.
Ethics oversight	No ethics approval was required for this study.

Note that full information on the approval of the study protocol must also be provided in the manuscript.

Field-specific reporting

Please select the one below that is the best fit for your research. If you are not sure, read the appropriate sections before making your selection.

☒ Life sciences ☐ Behavioural & social sciences ☐ Ecological, evolutionary & environmental sciences

For a reference copy of the document with all sections, see nature.com/documents/nr-reporting-summary-flat.pdf

Life sciences study design

All studies must disclose on these points even when the disclosure is negative.

Sample size	Size and length of the oligonucleotide pools was constrained by the capabilities and cost structure of the commercial synthesis provider to 170 nt and 11 293 individual sequences. This yielded at least 695 sequences per codec and code rate in the pools, such that stochastic effects on sequence dropout were precluded. To keep the sequence count per codec and code rate as homogeneous as possible, the size of the encoded data file was adjusted for some combinations of codec and code rates, thereby limiting the largest sub-pool to 1275 sequences.
Data exclusions	No data was excluded during analysis.
Replication	The experimental design included the replication of the workflow using a different synthesis process to investigate the effects on codec performance. Quantitation of initial pool concentration was performed using both absorbance and fluorescence (i.e., via Nanodrop and Qubit) to ensure the initial concentration was accurate and the sample purity was adequate. Quantitation of dilutions via qPCR were performed in duplicates, showing good agreement.
Randomization	To preclude bias from the spatial ordering of sequences on the synthesis chip, the sequence file sent to the commercial synthesis providers was randomized. As the sequences were also padded to identical lengths and featured identical amplification adapters, no discrimination of the sequences from individual codecs was possible during the experimental workflow, requiring no further randomization.
Blinding	Blinding was not required during the experimental data collection, as all sequences from all codecs and with all code rates were synthesized in parallel. This precluded any possibility of discrimination by the experimenter during amplification, dilution, and sequencing. During data analysis, no blinding was performed.

Reporting for specific materials, systems and methods

We require information from authors about some types of materials, experimental systems and methods used in many studies. Here, indicate whether each material, system or method listed is relevant to your study. If you are not sure if a list item applies to your research, read the appropriate section before selecting a response.

Materials & experimental systems

n/a	Involved in the study
☒	☐ Antibodies
☒	☐ Eukaryotic cell lines
☒	☐ Palaeontology and archaeology
☒	☐ Animals and other organisms
☒	☐ Clinical data
☒	☐ Dual use research of concern
☒	☐ Plants

Methods

n/a	Involved in the study
☒	☐ ChIP-seq
☒	☐ Flow cytometry
☒	☐ MRI-based neuroimaging

Plants

Seed stocks	*Report on the source of all seed stocks or other plant material used. If applicable, state the seed stock centre and catalogue number. If plant specimens were collected from the field, describe the collection location, date and sampling procedures.*
Novel plant genotypes	*Describe the methods by which all novel plant genotypes were produced. This includes those generated by transgenic approaches, gene editing, chemical/radiation-based mutagenesis and hybridization. For transgenic lines, describe the transformation method, the number of independent lines analyzed and the generation upon which experiments were performed. For gene-edited lines, describe the editor used, the endogenous sequence targeted for editing, the targeting guide RNA sequence (if applicable) and how the editor was applied.*
Authentication	*Describe any authentication procedures for each seed stock used or novel genotype generated. Describe any experiments used to assess the effect of a mutation and, where applicable, how potential secondary effects (e.g. second site T-DNA insertions, mosiacism, off-target gene editing) were examined.*
